# Adiposity-Dependent Regulatory Effects on Multi-tissue Transcriptomes

**DOI:** 10.1016/j.ajhg.2016.07.001

**Published:** 2016-09-01

**Authors:** Craig A. Glastonbury, Ana Viñuela, Alfonso Buil, Gisli H. Halldorsson, Gudmar Thorleifsson, Hannes Helgason, Unnur Thorsteinsdottir, Kari Stefansson, Emmanouil T. Dermitzakis, Tim D. Spector, Kerrin S. Small

**Affiliations:** 1 Department of Twin Research and Genetic Epidemiology, King’s College London, London SE1 7EH, UK; 2 Department of Genetic Medicine and Development, University of Geneva Medical School, Geneva 1211, Switzerland; 3 deCODE Genetics, Sturlugata 8, Reykjavik IS-101, Iceland; 4 School of Engineering and Natural Sciences, University of Iceland, Reykjavik 107, Iceland; 5 Faculty of Medicine, University of Iceland, Reykjavik 101, Iceland; 6 Institute for Genetics and Genomics in Geneva (iGE3), University of Geneva, Geneva C1211, Switzerland; 7 Swiss Institute of Bioinformatics, 1015 Lausanne, Switzerland

## Abstract

Obesity is a global epidemic that is causally associated with a range of diseases, including type 2 diabetes and cardiovascular disease, at the population-level. However, there is marked heterogeneity in obesity-related outcomes among individuals. This might reflect genotype-dependent responses to adiposity. Given that adiposity, measured by BMI, is associated with widespread changes in gene expression and regulatory variants mediate the majority of known complex trait loci, we sought to identify gene-by-BMI (G × BMI) interactions on the regulation of gene expression in a multi-tissue RNA-sequencing (RNA-seq) dataset from the TwinsUK cohort (n = 856). At a false discovery rate of 5%, we identified 16 *cis* G × BMI interactions (top *cis* interaction: *CHURC1*, rs7143432, p = 2.0 × 10^−12^) and one variant regulating 53 genes in *trans* (top *trans* interaction: *ZNF423*, rs3851570, p = 8.2 × 10^−13^), all in adipose tissue. The interactions were adipose-specific and enriched for variants overlapping adipocyte enhancers, and regulated genes were enriched for metabolic and inflammatory processes. We replicated a subset of the interactions in an independent adipose RNA-seq dataset (deCODE genetics, n = 754). We also confirmed the interactions with an alternate measure of obesity, dual-energy X-ray absorptiometry (DXA)-derived visceral-fat-volume measurements, in a subset of TwinsUK individuals (n = 682). The identified G × BMI regulatory effects demonstrate the dynamic nature of gene regulation and reveal a functional mechanism underlying the heterogeneous response to obesity. Additionally, we have provided a web browser allowing interactive exploration of the dataset, including of association between expression, BMI, and G × BMI regulatory effects in four tissues.

## Introduction

Obesity (MIM: 601665) is a global epidemic that has been robustly associated with a range of co-morbidities such as cardiovascular disease, insulin resistance, type 2 diabetes (T2D [MIM: 125853]), and increased risk of certain types of cancer.^1^, ^2^ However, at the individual level, co-morbidity development among obese individuals is heterogeneous, suggesting that genetics and adiposity might interact to mediate downstream disease and complex trait development.^3^ The existence of gene-by-adiposity interactions on complex traits is supported by twin studies demonstrating that BMI modifies the heritability of co-morbid traits such as blood pressure and insulin sensitivity.^4^, ^5^

Obesity manifests as excess adipose tissue and has a systemic effect on bodily function. BMI-associated genetic variants are enriched for hypothalamic processes,^6^ which suggests that the variants that cause obesity exert their effects primarily in the brain. In contrast, variants for many obesity co-morbid traits are primarily thought to regulate genes active in certain peripheral tissues, such as adipose, muscle, and the liver (insulin resistance),^7^ the heart and endothelial cells (QT-interval, which is predictive of cardiovascular disease),^8^ and adipose (body-fat distribution ),^9^ for example. Therefore, BMI could influence co-morbidity development by modifying gene expression in relevant peripheral tissues directly or by interacting with regulatory variants active in those tissues.

Gene-by-environment (G × E) interactions have been identified extensively in model organisms,^10^, ^11^, ^12^, ^13^ but identifying G × E interactions on complex traits in humans has proven to be difficult due to the need to obtain accurately measured environmental exposures and large sample sizes. In contrast, studies of G × E effects on the regulation of gene expression, often termed context-specific expression quantitative trait loci (eQTL) analyses, have been more successful and can capture environments that act on the cellular, tissue, or organismal level. Regulatory G × E effects have been discovered for various environments, including age and sex,^14^ in vivo and ex vivo treatment response,^15^, ^16^, ^17^, ^18^ and tissue and/or cell of residence.^19^, ^20^ Twin studies agnostic to the underlying environment have also identified G × E regulatory variants.^21^, ^22^ Most genetic variants identified through genome-wide association studies (GWASs) are thought to be regulatory variants; thus, utilizing gene expression to identify gene-by-environment interactions is a promising strategy for identifying factors that interact with disease-relevant regulatory variation.^22^ Because 60% of disease-associated eQTLs are tissue dependent, it is critical to study the genetic regulation of expression in the appropriate disease-relevant tissues.^23^

In this study, we considered BMI as a physiological environment and conduct a genome-wide search for gene-by-BMI (G × BMI) interactions on the regulation of gene expression by utilizing a multi-tissue (adipose, skin, whole blood, and lymphoblastoid cell lines [LCLs]) RNA-sequencing (RNA-seq) dataset including data collected from 856 healthy female twins. We first demonstrated that BMI has a pervasive effect on gene expression in peripheral tissues and that the strongest effects are observed in adipose tissue. We then identified 16 significant *cis* G × BMI interactions (false discovery rate [FDR] = 5%) on the regulation of gene expression in adipose tissue and we provide evidence of replicated examples in an independent adipose tissue cohort (deCODE, n = 754). The G × BMI regulatory interactions are adipose specific and are enriched for metabolic and inflammatory pathways. By extending the analysis in *trans* we were able to identify one *cis* G × BMI variant that regulates the expression of 53 genes in *trans* in a BMI-dependent manner. Regulatory interactions such as these will have increasingly important utility in characterizing the functionality and context specificity of genetic variation discovered via traditional GWAS approaches. To enable exploration of our results, we have developed an interactive website that allows researchers to model and plot data in real-time (see the Web Resources). Using this service, users can investigate genes and SNPs of interest for G × BMI effects and explore the relationship between BMI and exon-level expression in four separate tissues.

## Material and Methods

### Sample Collection

The study included 856 healthy female twins who are a part of the TwinsUK registry and are all of European ancestry. Punch biopsies of subcutaneous adipose tissue from a photo-protected area of the stomach adjacent and inferior to the umbilicus were obtained from consenting individuals. Skin from the punch biopsy was then dissected to separate it from adipose tissue, and both samples were weighed and immediately frozen with liquid nitrogen. Peripheral-blood samples were also collected as part of the study, and LCLs were generated via transformation of the B-lymphocyte fraction with Epstein-Barr virus (EBV). The European Collection of Cell Cultures agency performed the transformation process. All the procedures followed were in accordance with the ethical standards of the St. Thomas Research Ethics Committee (reference 07/H0802/84) at St. Thomas Hospital in London. Volunteers gave informed consent and signed a consent form before the biopsy procedure. Volunteers were supplied with an appropriate detailed information sheet regarding the research project and biopsy procedure by post before attending the biopsy.

### Genotyping and Imputation

Genome-wide SNP data for the TwinsUK individuals were generated as previously described.^6^, ^25^, ^26^ In short, TwinsUK samples were genotyped on a combination of platforms (HumanHap300, HumanHap610Q, and 1M-Duo Illumina arrays). Quality control and merging of the array datasets has previously been described in detail.^25^ The cleaned data were pre-phased with IMPUTE2 with no reference panel and were then imputed into the 1000 Genomes phase 1 reference panel (interim, data freeze accessed November 10, 2010; the 1000 Genomes Project Consortium 2012).^27^ Variants with an INFO score >0.8 on all platforms and a MAF >5% were retained for analysis.

### Phenotype Collection

Height, weight, and visceral fat volume were measured at the time of biopsy. Visceral fat volume was measured via dual-energy X-ray absorptiometry (DXA; Hologic QDR 4500 Plus), according to the standard manufacturer’s protocol.

### RNA-Seq

RNA-seq data were generated as previously described.^22^ In brief, samples were prepared for sequencing with the unstranded Illumina TruSeq sample preparation kit and sequenced on a HiSeq 2000 machine. The 49 bp paired-end reads were aligned to the UCSC Genome Browser GRCh37 reference genome with the Burrows-Wheeler Aligner.^28^ GENCODE v.10 was used to annotate genes. Samples were excluded if they failed to have more than 10 million reads map to known exons or if the sequence data did not correspond to actual genotype data.

### Exon Quantification and Normalization

To quantify exons, all overlapping exons of a gene were merged into one meta-exon. We counted reads as mapping to a given meta-exon if either of its start or end coordinates overlapped a meta-exon boundary. All read-count quantifications were corrected for variation in sequencing depth between samples by normalizing the number of reads to the median number of well-mapped reads. We only used exons that were quantified in more than 90% of the individuals. Exon expression values were rank-based inverse normal transformed for downstream analysis.

### Transcriptome-wide Association Analysis

To determine whether expressed exons were associated to a phenotype of interest (BMI/visceral fat), each exon was treated as a quantitative trait in a linear mixed-effects model implemented with the lme4 package.^29^ Phenotypic data were treated as continuous independent traits. A full model with the phenotype fitted (Model 1) was compared to a null model in which the same model was fitted but the phenotype (BMI) was omitted. These models were compared with a one-degree-of-freedom ANOVA. All known technical variables were included as covariates in the model. To model the twin structure in our data, we included two multi-level indicator variables, termed family and zygosity, as random effects in the model. These variables are coded as follows: if individuals i and j are co-twins, we code family_i_ = family_j_, if individuals i and j are monozygotic co-twins, we code zygosity_i_ = zygosity_j,_ and if they are dizygotic co-twins, we code zygosity_i_ ≠ zygosity_j_. The zygosity term captures the increased genetic relatedness within an MZ twin pair as compared to a DZ twin pair. If individuals i and j are unrelated, family_i_ ≠ family_j_ and zygosity_i_ ≠ zygosity_j_. We estimated a FDR within each tissue by using the package “qvalue”^30^ to obtain q values that correspond to a FDR of 5%. Model 1 is as follows:

(Model 1) $$y_{i}∼\text{Xβ}+\text{Zu}+\varepsilon ,$$

where *y*_*i*_ is the *i*^th^ non-PEER (probabilistic estimation of expression residuals)-corrected exon expression vector. X is a design matrix of fixed effects: phenotype (BMI or visceral fat), age, age squared, mean GC content, and insert size mode. Z represents all random effects: primer index, batch (blood only), family, and zygosity. *ε* is a residual error term representing unaccounted for variation in expression.

### *cis* G × BMI Interaction Discovery

Expression residuals corrected for family structure and technical variables and 50 PEER^31^ factors were used to identify G × BMI interactions in all four tissues. Each exon was tested for a SNP × BMI interaction (Model 2) with all SNPs within 1 Mb of the transcription start site. The interaction test was implemented with the “modellinear_cross” function in the Matrix eQTL R package.^32^ Model 2 is as follows:

(Model 2) $$y∼\text{I}+\text{β}A+{\text{β}A}^{2}+\text{β}P+\text{β}SNP+\text{β}P\;\times \;SNP+\varepsilon ,$$

where *y* = expression, I = intercept, *A* = age, *A*^2^ = age squared, *P* = phenotype (BMI), *SNP* = allele dosage, and *ε* = residual error.

### Correction for Hidden Confounders

A common approach when performing eQTL mapping has been to adjust expression profiles for unknown latent variables. The aim is to capture hidden factors that confound gene expression measurements, such as unmeasured technical variables (e.g., batch effects). However, these methods can also capture biological factors, and thus, depending on the analysis performed, might not be appropriate. We utilized PEER, a Bayesian method similar to surrogate variable analysis, which has been shown to increase the ability to detect genetic and interaction effects threefold.^31^ We calculated latent factors by applying PEER to expression residuals that had been corrected for family structure and technical variables. PEER was run using no additional covariates and accounting for mean expression. In adipose tissue, three of the first five PEER factors were significantly correlated to BMI (**|**r_max_**|** = 0.52, p = 1.2 × 10^−51^, Figure S1). We used PEER-corrected data for *cis* G × BMI interaction analysis, but not for direct association of BMI with expression levels or for *trans* G × BMI interaction analysis (see below). Removing BMI-associated PEER factors maximizes the ability to discover interactions with BMI by minimizing model co-linearity (Figures S1 and S2). It has also been demonstrated that removing latent factors, even those highly correlated with the environment of interest, results in the discovery of significantly more interactions on gene expression.^20^

### FDR Estimation

The FDR for calling an interaction significant was estimated based on an approximated permutation strategy as previously described and implemented.^33^, ^34^ Expression residuals were calculated in which all main effects (BMI, SNP, and age) were regressed out. Expression values were permuted for each exon while preserving genotype structure. The interaction-term (BMI × SNP) significance was calculated for each exon. All p values were stored to calculate a genome-wide FDR. Because genes vary in the number of exons they are composed of, separate FDRs were calculated by classifying genes based on a similar number of exons, determined from the distribution of exons expressed in each tissue. A 5% FDR was calculated by computing the ratio of permuted test statistics more significant to the observed interactions, divided by the number of permutations performed (n = 100). All interactions with a corrected FDR p value <0.05 were classified as significant and taken forward for replication.

### *trans* G × BMI Discovery

Given that *cis* effects are enriched for *trans* effects and we were underpowered to perform a genome-wide *trans*-interaction analysis, we utilized a two-step procedure to identify *trans* G × BMI interactions in adipose tissue. We first repeated the *cis* G × BMI scan as described above, using expression residuals corrected for family structure and known technical variables but not corrected for PEER factors. PEER correction removes broadly acting variance components, including the effects of multi-gene *trans*-regulators, and is therefore not appropriate for *trans* eQTL analysis. The resulting four significant non-PEER-corrected *cis* G × BMI variants (rs1464171, rs3851570, rs113368712 and rs35662778; FDR = 5%) were then tested for *trans* G × BMI effects. *Trans* effects were tested for all genes at a distance greater than 5 MB from the variant or on a different chromosome. *Trans* G × BMI significance was assessed with a strict Bonferroni correction, accounting for testing four variants against 116,643 exons and corresponding to a p value threshold of 1.1 × 10^−7^.

### deCODE Replication Cohort

The replication cohort consists of 754 RNA-seq subcutaneous adipose samples obtained from Icelandic individuals, all of whom had imputed genotypes, as previously described.^35^ The sample set is comprised of 333 males and 421 female participants with an age 47 ± 14 (mean ± SD) and BMI 30 ± 6.6. RNA-seq reads were aligned to *Homo sapiens* build 38 (UCSC Genome Browser) with TopHat^36^ v.2.0.12 with a supplied set of known transcripts in GTF format (RefSeq, NCBI). TopHat was configured such that it first attempts to align reads to the provided transcriptome, then, for reads that do not map fully to the transcriptome, it attempts to map them onto the genome. Overlapping exons were merged into one meta-exon. Fragments were counted if they mapped to a meta-exon and if either read in the pair had the start or end (aligned) coordinate overlapping a meta-exon boundary. We excluded exons that had zero fragment counts for more than 90% of individuals. Counts were normalized for number of reads mapped and exon lengths and were rank normal transformed. The following covariates were used to correct for technical differences in the RNA experiments: average fragment length, exonic rate, number of genes detected, number of mapped read pairs, number of alternative alignments, and percentage of reads originating from coding bases (PCT), along with 50 inferred hidden factors which were evaluated with PEER. The technical RNA-seq quality metrics were gathered with the CollectRnaSeqMetrics tool in Picard v.1.79 and RNA-SeQC v.1.1.6.^37^

### Replication Analysis

The 16 *cis* G × BMI interactions (FDR = 5%) were taken forward for replication in deCODE. 13 genes were available in the deCODE dataset. The two datasets were called with different genome builds and gene annotation sets, preventing simple mapping of meta-exons. We thus used liftover to map the TwinsUK meta-exon coordinates from GrCh37 to GrCh38 (UCSC Genome Browser) and defined corresponding meta-exons as those with more than a 90% overlap in length in both annotation sets. This strategy identified corresponding meta-exons for eight genes (*CHURC1* [MIM: 608577], *CIDEA* [MIM: 604440], *ZNF117* [MIM: 194624], *PEPD* [MIM: 613230], *ANXA5* [MIM: 131230], *HLA-DQB2* [MIM: 615161], *IFNAR1* [MIM: 107450], and *SCFD2).* An additional three genes (*ADH1A* [MIM: 103700], *SPAG17* [MIM: 616554], and *ERV3-1* [MIM: 131170]) had a partial overlap, ranging from 33%–85%, with a deCODE meta-exon Two genes (*PHACTR3* [MIM: 608725] and *CAST* [MIM: 114090]) had minimal overlap of 4% with a deCODE meta-exon, indicating the meta-exon does not represent an analogous quantification. Given that multiple TwinsUK *PHACTR3* meta-exons were significant, and the second most significant TwinsUK meta-exon (chr20: 58,349,298–58,349,545, p value = 1.7 × 10^−8^ in TwinsUK) exactly overlapped a deCODE meta-exon, we used the meta-exon corresponding to the second TwinsUK signal for the replication analysis of *PHACTR3*. Exact meta-exon coordinates are provided in Table S1. Given the low annotation overlap at *CAST* (4%), we tested all *CAST* exons for replication and corrected for the number of exons tested in deCODE (30). The three genes unavailable for replications were *RP11-71E19.1*, which was not present in the RefSeq gene annotation used by deCODE, *POU6F2* (MIM: 609062), which did not pass quality control in deCODE (low expression), and *SIK1* (MIM: 605705), which was quantified, but the corresponding SNP rs12482956 and proxies were unavailable in the deCODE imputation.

### Integration with GWAS

Overlap of the 16 G × BMI lead SNPs with GWAS variants was determined by searching the National Human Genome Research Institute (NHGRI) database (accessed June 19, 2015) for each SNP or proxy SNPs (r^2^ > 0.6). The database was filtered to include only genome-wide significant loci. We tested the overlap identified at *ADH1A* for colocalization by implementing the regulatory trait concordance (RTC) method.^53^ The RTC score assesses evidence for causality by testing whether the effect of a GWAS SNP abrogates the effect of an eQTL while accounting for the local linkage disequilibrium (LD) structure of the locus. All SNPs with MAF > 5% were in the window 100.1–100.6 Mb, a 250 kb window centered on the index SNP tested. For each of the 1,129 variants, we fitted an interaction (Model 3) and main effect (Model 4) model:

(Model 3) $$ADH1A\_\text{expression}∼\text{BMI}\times {\text{SNP}}_{\text{N}}+\text{BMI}\times \text{rs}1693457$$

and

(Model 4) $$ADH1A\_\text{expression}∼{\text{SNP}}_{\text{N}}+\text{BMI}\times \text{rs}1693457\text{,}$$

where SNP_N_ is SNP_1_–SNP_1,129_.

The results were then ranked by increasing significance of the interaction p value (BMI × rs1693457), and RTC scores were calculated as follows:

(Equation 1) $$\text{RTC}=\frac{N_{\text{SNPs}}-Rank_{\text{GWAS}\_\text{SNP}}}{N_{\text{SNPs}}}$$

where *N*_SNPs_ = number of SNPs tested.

*Rank*_GWAS_SNP_ = the rank of the GWAS SNP in the full list of ordered test statistics.

### Tissue Specificity and eQTL π_1_ Analysis

To determine the tissue specificity of identified G × BMI interactions and whether they are enriched for main-effect eQTLs, π_1_ analysis was performed.^30^ π_0_ is a measure for estimating the proportion of true null hypotheses. 1 − π_0_ = π_1_ can therefore be used to measure the proportion of significant (true) associations. The significant exon-SNP pairs from adipose tissue were matched to the same exon-SNP pair in each of the other tissues, and a π_1_ value was estimated with the R package “qvalue”^30^. The same method was used to determine whether the significant G × BMI interactions are enriched for main-effect eQTLs.

### Gene Set Enrichment

Both *cis* and *trans* gene set enrichment analysis data were analyzed through the use of QIAGEN’s Ingenuity Pathway Analysis (IPA). Input consists of the gene list of interest, and to correctly control for background genes, we used all genes expressed in our adipose tissue expression datasets as a reference to compare against. Benjamini-Hochberg corrected p values were calculated as specified by Ingenuity. The macrophage-enriched metabolic network (MEMN) gene membership was tested for G × BMI enrichment via π_1_ analysis, as above.

### *Trans*-Network Mediation Analysis

Significant mediation was determined by computing Sobel’s test statistic.^38^ To calculate the mediation score, the following four models and equations were implemented:

(Model 5) $$y∼{\text{β}}_{1}A+{\text{β}}_{2}A^{2}+{\text{β}}_{3}P+{\text{β}}_{4}G+{\text{β}}_{5}P\times G+\varepsilon ,$$

(Model 6) $$y∼{\text{β}}_{1}E+{\text{β}}_{2}A+{\text{β}}_{3}A^{2}+{\text{β}}_{4}P+{\text{β}}_{5}G+{\text{β}}_{6}P\times G+\varepsilon ,$$

(Equation 2) $$\text{Mediation}\_\text{score}=\frac{{\text{β}}_{\text{M}5\_5}-{\text{β}}_{\text{M}6\_6}}{{\text{β}}_{\text{M}5\_5}},$$

and

(Equation 3) $$Z=\frac{{\text{β}}_{\text{M}5\_5}\times {\text{β}}_{\text{M}6\_6}}{\sqrt{{\text{β}}_{\text{M}6\_6}^{2}\times S_{\text{M}5\_5}^{2}+{\text{β}}_{\text{M}5\_5}^{2}\times S_{\text{M}6\_6}^{2}}},$$

where *y* = *trans* gene expression, *A* = age, *A*^2^ = age squared, *E* = *cis* gene expression, *P* = BMI, and *G* = *cis* genotype. M5_5 is the interaction coefficient from Model 5. M6_6 is the interaction coefficient from Model 6. *S*^2^ is the SE of each interaction coefficient.

By conditioning on *cis* gene expression (the mediator, *E*) we can determine whether each individual interaction detected in *trans* is regulated in *cis* or is independent by quantifying *ΔβP × G* (Equation 2). We can test the significance of this change by using Sobel’s test statistic (Equation 3).

### Roadmap Epigenomics Functional Element Enrichment Analysis

To investigate enrichment of cell-type-specific enhancers, HaploReg v.4 was utilized.^39^ HaploReg takes a list of SNPs and uses a binomial test for enhancer enrichment in different cell types based on the SNP of interest (G × BMI SNP) and any SNPs that are in high LD with the lead SNP (r^2^ > 0.8). These SNPs are then compared to a background that consists of the common variants (5%) obtained from the 1000 Genomes Project. Enhancer annotation was obtained from the ChromHMM 15-state model.

### Interactive Website

The website was designed with RMarkdown, custom CSS, and a shiny backend server for real-time statistical analysis. All models implemented are those described above, with additional options given to users to allow the choice of covariate inclusion and/or PEER correction.

## Results

### BMI Has a Pervasive Effect on Gene Expression

To characterize the effect of BMI on the transcriptome, we utilized a multi-tissue RNA-seq dataset including data from 856 healthy female twins in the TwinsUK cohort.^22^ Participants were 38–84 years old (median = 60) with a BMI range of 16–47 (median = 25) at the time of biopsy (Figure S3). As described previously,^22^ after quality control, RNA-seq data and imputed genotypes from the 1000 Genomes phase 1 reference panel were available for 720 subcutaneous adipose tissue samples, 672 skin samples, 765 LCLs, and 368 whole-blood samples. All analysis was based on exon-level quantifications with 16,149 to 18,229 genes expressed per tissue (Table S2).

**Figure 1:**
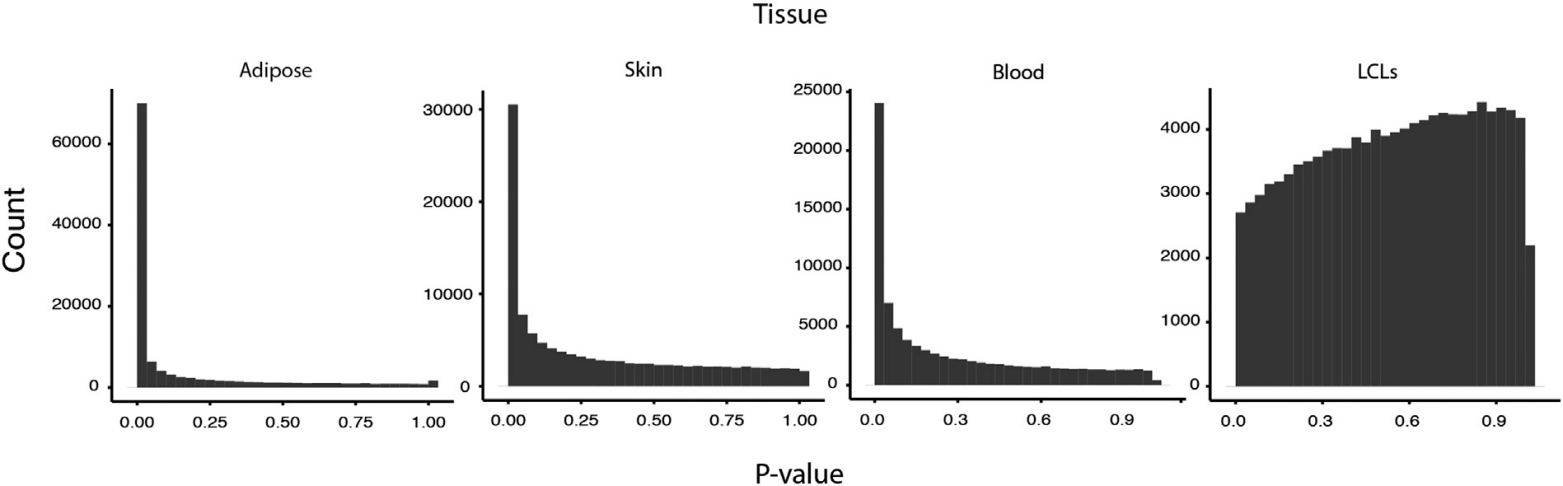
BMI Is Highly Associated with Gene Expression in Multiple Tissues p value distribution of association between BMI and expression of all measured exons in each tissue.

We first assessed the extent of association between BMI and gene expression in all four tissues (adipose, skin, blood, and LCLs). BMI has a strong influence on expression in each of the primary tissues, but little to no effect in LCLs; with a FDR of 5%, 16,818 genes, 9,216 genes, 6,640 genes, and zero genes had at least one exon associated with BMI in adipose, skin, blood, and LCLs respectively (Figure 1). We assessed the tissue specificity of the associations by using π_1_ estimates (Figure S4). Approximately half of the associations detected in adipose were observed in the other two primary tissues (skin π_1_ = 0.53, blood π_1_ = 0.54), whereas adipose captured over 75% of the associations detected in skin (π_1_ = 0.78) and blood (π_1_ = 0.76). There was no enrichment for shared effects in LCLs (π_1_ = 0 for all tissues), which is consistent with a lack of LCL expression and whole-body trait associations seen for multiple traits,^21^ suggesting the transformation process or subsequent cell culture has removed the in vivo physiological environmental effects captured by profiling primary tissue. Our estimates of a pervasive and tissue-specific influence of BMI on expression are consistent with previous estimates from microarrays^40^ and confirm that BMI acts as a strong physiological influence on gene expression. Full summary statistics for the association between BMI and exon-level expression in all four tissues can be found at our website (see Web Resources).

### Identification of *cis* G × BMI Regulatory Variants

**Figure 2:**
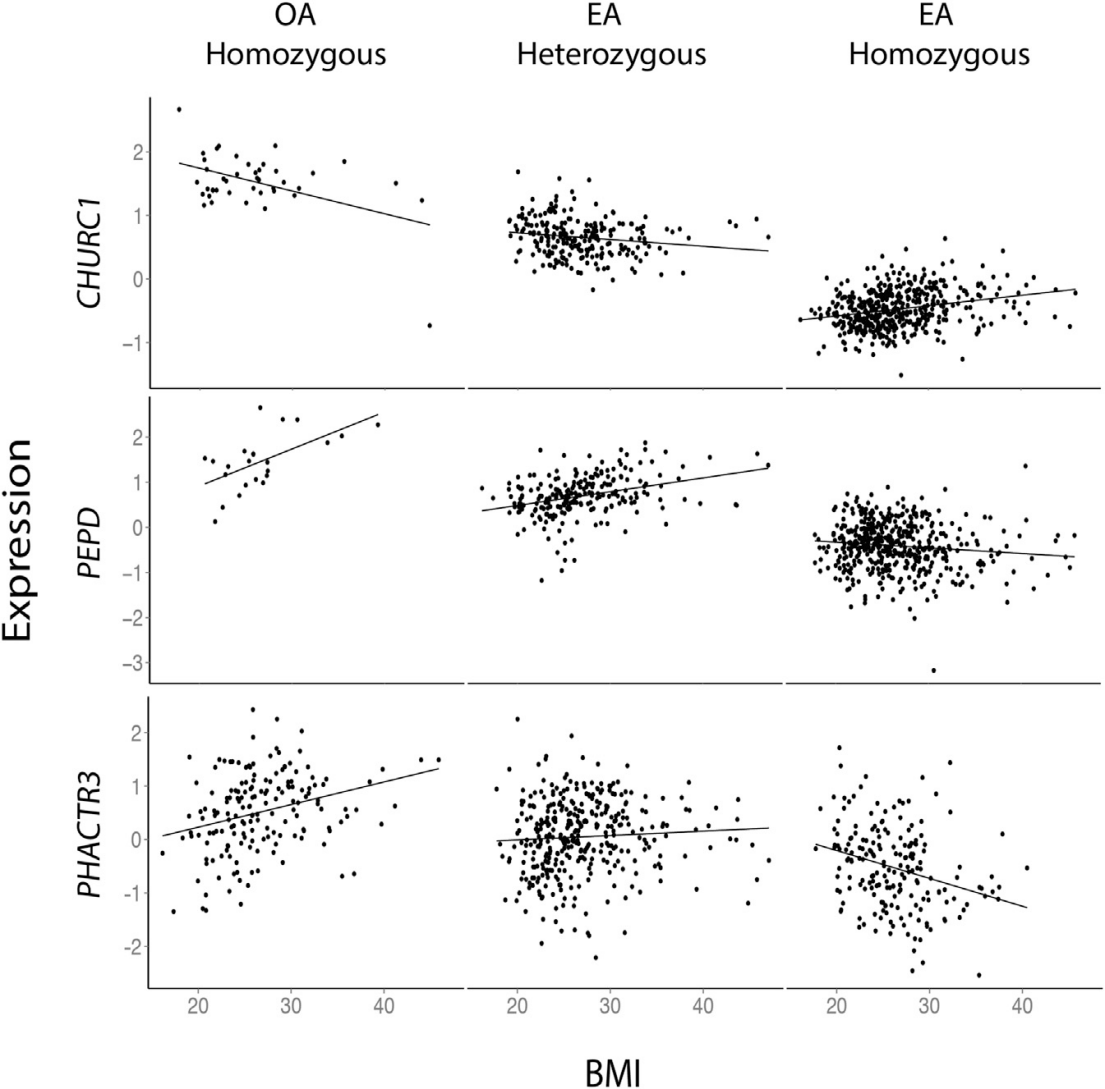
Example of Three FDR = 5% Significant Adipose *cis* G × BMI Regulatory Interactions The vertical axis represents expression of a given gene and the horizontal axis represents BMI. Each point represents an individual. Each plot is split on allele dosage to show how the relationship between expression and BMI is dependent on genotype (OA, other allele; EA, effect allele; corresponding variants and alleles are listed in Table 1). A change in the slope of the association between BMI and expression association across genotypic classes indicates a genotype-dependent response to BMI, or G × BMI interaction. For example, *CHURC1* expression decreases with BMI in individuals homozygous for the effect allele, but increases with BMI in other allele homozygotes regardless of the mean change in gene expression (main-effect eQTL).

To identify BMI-dependent regulatory effects, we performed a global *cis* scan for G × BMI eQTLs in each tissue by using PEER-corrected expression residuals. Significant G × BMI effects (FDR = 5%) were called with a per-tissue FDR, determined by permutation (see Material and Methods). Our FDR method ensures we penalize against genes with more exons given that, statistically, they are more likely to show association by chance. We identified 16 G × BMI regulatory effects in adipose (Table 1, Table S3, Figure 2) and none in the other three tissues. The 16 genes regulated by a G × BMI effect in adipose are *ADH1A*, *ANXA5*, *CAST*, *CHURC1*, *CIDEA*, *ERV3-1*, *HLA-DQB2*, *IFNAR1*, *PEPD*, *PHACTR3*, *SCFD2*, *SPAG17*, and *ZNF117*. 12 of the 16 G × BMI variants also had a significant main effect (eQTL) on adipose expression when tested without the interaction term (Table 1, Table S3).

**Figure 3:**
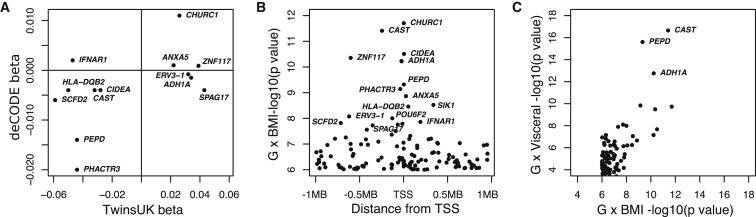
Replication, Location, and Validation of *cis* G × BMI Interactions (A) Comparison of G × BMI interaction coefficients (FDR = 5%) in the TwinsUK discovery (horizontal axis) and deCODE replication dataset (vertical axis). 9 out of 13 interactions show a consistent direction of effect across cohorts. (B) The lead G × BMI SNPs all cluster at the TSS. Location of the lead SNPs for the 127 G × BMI interactions with p < 10^−6^ are plotted with respect to the TSS of the corresponding gene. Lead SNPs for the 26 significant G × BMI interactions (FDR = 5%) are labeled with the corresponding gene name. (C) Comparison of p values of G × BMI interaction and G × visceral-fat interaction for the same SNP gene pairs. All 127 interactions with G × BMI p < 10^−6^ are plotted. All 16 G × BMI (FDR = 5%) show a G × visceral-fat interaction, three of which increased 2–5 orders of magnitude in despite the smaller sample size in the visceral fat analysis (n = 682).

We sought replication in an independent dataset of subcutaneous adipose tissue biopsies (Icelandic cohort, deCODE genetics, n = 754), which included quantification of 13 of the 16 genes. Because TwinsUK and deCODE used different alignment strategies, genome builds, and gene annotation versions, some exons did not directly map between the two datasets. We identified corresponding exons with a greater than 90% overlap in annotation in both datasets for nine genes (Table S1). Of these nine, three were replicated and eight showed a consistent direction of effect between the studies (Figure 3A). The replicated genes were *PEPD* (p_TwinsUK_ = 4.8 × 10^−10^, p_deCODE_ = 4.2 × 10^−6^), *PHACTR3* (p_TwinsUK_ = 1.6. × 10^−8^, p_deCODE_ = 1.1 × 10^−4^), and *CHURC1* (p_TwinsUK_ = 2.0 × 10^−12^, p_deCODE_ = 8.5 × 10^−4^) (Table 1). Another four genes showed partial overlap with a deCODE exon: *ERV3-1* (85% overlap), *SPAG17* (69% overlap), *ADH1A* (33% overlap), and *CAST* (4% overlap). None of these replicated and only *CAST* had a consistent direction of effect, with p = 0.053. Given the low annotation overlap at *CAST* (4%), we examined all other *CAST* exons in deCODE, and the exon in chr5: 96,076,448–96,076,487 was associated in a consistent direction at nominal significance (p = 0.001, p = 0.03 corrected for 30 *CAST* exons). Overall, the replication demonstrates the robustness of our findings and the discovery and independent replication of regulatory variants whose effects are influenced by adiposity.

### Tissue Specificity of G × BMI Regulatory Effects and Expression

The G × BMI regulatory effects are highly tissue specific. None of the 16 G × BMI interaction effects are present in the other TwinsUK tissues (Table S3). Although 11 G × BMI regulated genes showed multi-tissue expression, five genes (*PHACTR3*, *ADH1A*, *RP11-71E19.1*, *POU6F2*, and *SPAG17*) have adipose-specific expression within TwinsUK but no detectable expression in blood, skin, or LCLs. The G × BMI variants are enriched for tissue-specific regulatory potential; we tested the 16 variants for enrichment in enhancer activity in the 127 Roadmap Epigenomics cell types and found an enrichment only in purified adipocytes (mesenchymal-stem-cell-derived adipocytes, p = 0.028). Nine G × BMI variants disrupt specific or multiple transcription factor binding motifs, and eight SNPs or their proxies (r^2^ > 0.8) have a significant evolutionary GERP conservation score (Table S3). The tissue specificity of G × BMI effects, expression of the genes they act on, and regulatory annotation of the G × BMI variants highlights the importance of studying gene regulation in the appropriate tissue for a disease of interest.

### G × BMI Regulatory Variants and Genes Link to Related Traits

We next investigated links between the G × BMI genes and variants and related traits. *CAST* (calpastatin) is a ubiquitously expressed endogenous inhibitor of the calpains (calcium-dependent cysteine proteases). The calpastatin-calpain system has been extensively implicated in cardiac remodeling and heart failure and has been proposed as a potential therapeutic target for heart disease.^41^ *CAST* is implicated in modulating the immune response in certain cell types,^42^ including inhibition of macrophage hyperactivation under inflammatory conditions,^43^ which could be linked to the varying degrees of systemic inflammation seen in obese individuals.

Another intriguing signal is at *PEPD* (peptidase D). *PEPD’s* function is the recycling of proline, and it has also been shown to be essential for collagen production. Variants intronic to *PEPD* are associated with T2D, adiponectin, triglyceride levels, and fasting insulin;^44^, ^45^, ^46^, ^47^ however, the lead GWAS SNPs are in low LD with the G × BMI regulatory variant (r^2^ = 0.145, D^′^ = 1). *PEPD*’s link to T2D and adiponectin, although not fully understood, is interesting in the light of BMI’s relationship with T2D development and adiponectin regulation.

*CIDEA* (cell death activator) function has been studied extensively in model organisms, and *CIDEA*-knockout mice show higher basal metabolic rates, lipolysis, and higher core body temperatures.^48^ Mice without functional copies of *CIDEA* are resistant to both diet-induced obesity and T2D.^49^, ^50^ Similar evidence has been observed in humans.^51^ In our data, *CIDEA* expression has a strong negative correlation with BMI (p value = 8.1 × 10^−54^). However, individuals with the minor allele of rs7505859 show the opposite relationship (*CIDEA* is positively correlated with BMI). Again, this is an interesting relationship in the light of BMI’s contribution to T2D development.

We integrated the 16 G × BMI variants with the NHGRI database of GWASs to determine whether the G × BMI regulatory effects are linked to common traits or disease. A proxy SNP (rs1229977, r^2^ = 0.63) for the G × BMI variant regulating *ADH1A* (alcohol dehydrogenase 1A), rs1693457, is associated with esophageal cancer (MIM: 133239) in a large GWAS.^52^ The GWAS association at this locus covers several ADH family genes; however, we show that, in addition to the G × BMI effect, rs1693457 is a main-effect eQTL (β = −0.89, p = 2.9 × 10^−41^) for *ADH1A*. To formally test for colocalization between the regulatory variant and the GWAS signal, we implemented the regulatory trait concordance method (RTC).^53^ The esophageal cancer GWAS SNP (rs1229977) had an RTC score of 0.98 when tested with either the interaction or main-effect model, indicating that the regulatory and GWAS signals are tagging the same underlying variant. There is prior evidence for a G × E interaction at rs1229977—the association to esophageal cancer is modified by alcohol consumption.^52^ Given the complicated links between BMI, alcohol intake, and smoking, it is possible that BMI is acting as a proxy for correlated external environmental factors at this locus, which could also explain the lack of replication of the *ADH1A* G × BMI effect in the Icelandic data, given that external environments can vary between countries.

### Detection of *trans* G × BMI Effects in Adipose Tissue

**Figure 4:**
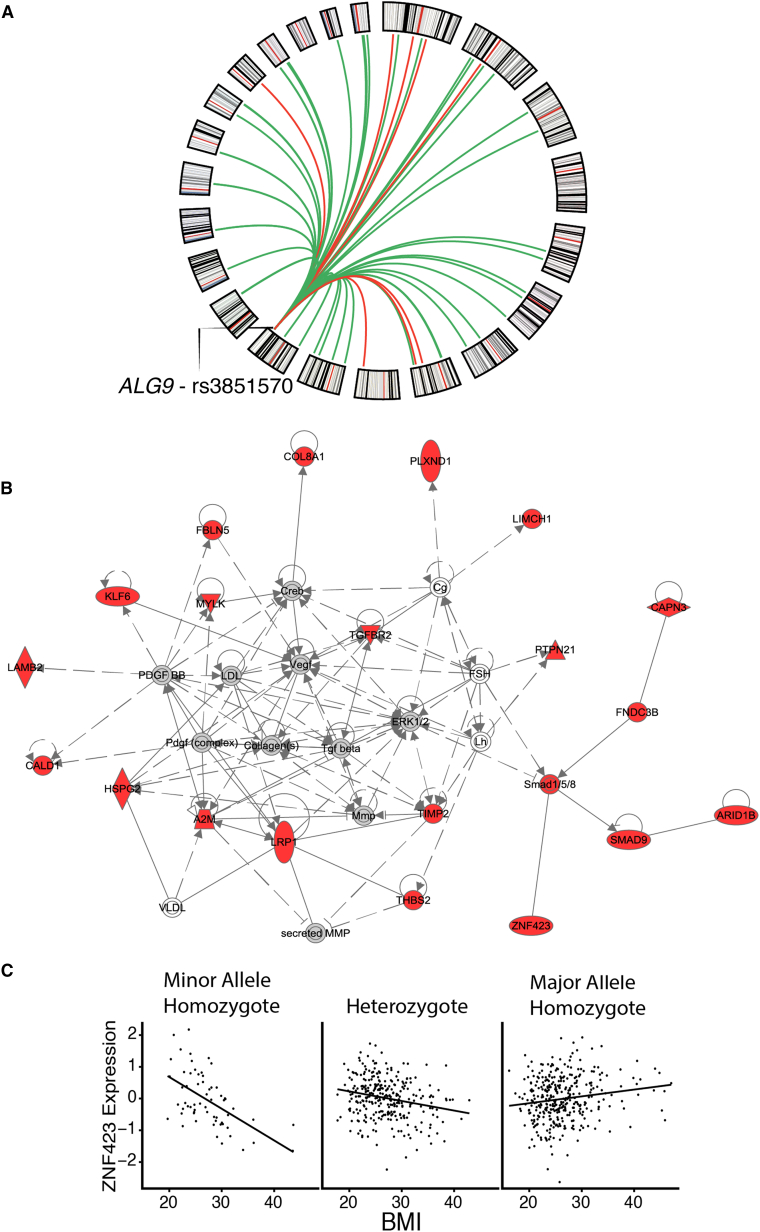
rs3851570 Has a *trans* G × BMI Effect on 53 Genes (A) Circos plot displaying the location of rs3851570 and *trans* G × BMI associations. Each line represents a *trans* association originating from rs3851570. Green lines indicate a positive beta, and red lines represent a negative beta. The outer circle delineates chromosomes, including cytogenetic bands. (B) *Trans* genes are enriched for membership in a cardiovascular disease network (IPA). Plot displays all genes in the cardiovascular disease network. Genes highlighted in red are present in the *trans*-network, and non-*trans* network genes are shown in gray. Lines between genes represent their regulatory relationship. Solid lines represent direct evidence of interaction, while dashed lines indicate indirect interaction evidence. The shape of each node denotes the gene's primary known function (protein kinase, transcription factor, complex, etc). (C) A signal plot of the most significant G × BMI *trans*-association to rs3851570, at the gene *ZNF423*. The vertical axis represents expression of *ZNF423* and the horizontal-axis is BMI. Each point represents an individual; individuals are grouped into the three boxes according to their allele dosage for SNP rs3851570.

Evidence from model organisms suggests that *trans* (distal) regulatory variants are more likely to mediate the effects of the environment and are thus strong candidates for G × E interaction effects.^10^ In contrast to *cis* eQTLs, *trans* eQTLs have been difficult to identify in humans because of smaller effect sizes and the increased burden of multiple testing in a genome-wide scan. Given that *cis* eQTLs are enriched for *trans*-eQTL effects,^54^ we utilized a two-step strategy to identify *trans* G × BMI interactions in adipose tissue, first identifying *cis* G × BMI interactions and then testing the identified *cis* G × BMI variants for *trans* G × BMI effects with all adipose-expressed exons. For *trans* discovery, both steps were performed with non-PEER-corrected expression residuals because correction for latent factors can remove broadly acting *trans* effects. This strategy identified four *cis* G × BMI variants (Table S4), one of which, rs3851570 (*cis* G × BMI interaction on *ALG9* expression [MIM: 606941], p value = 2.0 × 10^−8^), is associated with 53 genes in *trans* (Bonferroni corrected p value threshold of p < 1.1 × 10^−7^) (Figure 4A, Table S5, Figure S5).

IPA of the 53 genes in the *ALG9* G × BMI *trans*-network revealed enrichment for inhibition of matrix metalloproteases (Benjamini-Hochberg corrected p = 3.6 × 10^−8^), oxidative phosphorylation (Benjamini-Hochberg corrected p = 3.1 × 10^−4^), and gene membership of a cardiovascular disease network (Figure 4B), indicating that BMI-dependent regulatory effects at rs3851570 have a wide-ranging role in metabolism and structural remodeling of adipose tissue. *ALG9* itself catalyzes lipid-linked oligosaccharide assembly in the N-glycan biosynthesis pathway. Statistical mediation analysis supports a mediator role of *ALG9* expression in regulation of the *trans* G × BMI network (Sobel’s mediation p value ≤ 0.001, Table S6) (see Material and Methods). Given that *ALG9* has no known regulatory role on transcription, the regulation of the *trans* genes most likely functions via regulation of signaling cascades or other complex indirect processes. We note that the most significant *trans* G × BMI gene is the transcription factor *ZNF423 (*p = 8.2 × 10^−13^) (Figure 4C). *ZNF423* regulates pre-adipocyte determination and expression of *PPARG* (MIM: 601487), a master regulator of adipocyte differentiation,^55^ and could be a candidate for mediating the widespread *trans* effect, although this would require further investigation. We investigated the *trans* G × BMI effect in a second population by testing rs3851570 against all measured exon expression amounts (n = 168,951) in the deCODE dataset. Although the 53 *trans* genes did not replicate in the deCODE dataset, transcriptome-wide we see a significant enrichment for low p values (π_1_ = 0.14), demonstrating that rs3851570 has broad effects on adipose gene expression in a BMI-dependent manner in multiple populations. This is consistent with previous studies, wherein transcriptome-wide regulatory behavior of the multi-gene *trans* variants replicate across cohorts but the top genes associated with such *trans* networks do not.^23^

### General Properties of *cis* G × BMI Regulatory Effects and Variants

To investigate the common properties of G × BMI regulatory effects and the genes they act upon, we next focused on the 127 adipose G × BMI *cis* effects that passed a relaxed threshold of p < 1.0 × 10^−6^ (Table S7). All 127 G × BMI interactions show an opposite direction of effect between expression and BMI in the two-homozygote classes (for example, *PHACTR3*, Figure 2). This high prevalence of opposing direction of effect most likely reflects our power to detect opposing effects rather than the true distribution. Consistent with main-effect eQTL variants, G × BMI variants are enriched for proximity to the transcription start site, most noticeably when looking at genome-wide significant G × BMI variants (median distance, 38 kb) (Figure 3B). The 127 G × BMI variants show significant adipose main effects when tested without an interaction with BMI (number of eQTLs (FDR = 1%): adipose = 20, blood = 8, LCLs = 10, and skin = 10) (Figure S6). Whereas main-effect eQTL enrichment was observed in other tissues, the G × BMI effect is adipose-tissue specific, with π_1_ = 0.038 in skin and π_1_ = 0 in blood and LCLs. G × BMI variants are not directly associated with BMI, and no significant enrichment was seen in a BMI GWAS of 339,224 individuals^6^ (π_1_ = 0), suggesting the G × BMI effects are not the result of indirectly measured gene-by-gene effects.

### G × BMI Are Enriched for Key Metabolic Processes

To elucidate the biological consequences of BMI-dependent regulation, we investigated the 127 genes regulated by a G × BMI interaction at p < 1.0 × 10^−6^ for functional enrichment by using IPA. The 127 genes are enriched for key metabolic processes, including LXL and RXR activation (p = 6.1 × 10^−3^) and uptake of cholesterol (p = 9.1 × 10^−5^) (Table S8). *LXL* and *RXR* are part of the insulin-signaling pathway and are the target of several widely used T2D medications.^56^ Additionally, the 127 genes are enriched for the antigen-presentation pathway (p = 2.0 × 10^−4^) and quantity of macrophages (p = 5.8 × 10^−3^). We do not see evidence for G × BMI effects in the BMI-associated MEMN of co-expressed adipose genes.^40^, ^57^ The 553 MEMN genes show no enrichment for G × BMI effects (π_1_ = 0.021), and there is not a significant overlap between the 127 G × BMI genes and MEMN genes (binomial p = 0.78). However, we do note that of the four G × BMI genes in the MEMN (*ICAM3* [MIM: 146631], *TBX3* [MIM: 601621], *CIDEA*, and *ADH1A*), two do have genome-wide-significant G × BMI effects (*CIDEA* and *ADH1A*).

### BMI Accurately Captures the Effects of Adiposity

BMI is an easily measured anthropometric trait that is commonly used as a surrogate for overall adiposity. It has been noted that BMI can potentially misclassify an individual as obese, for example, when a subject has a large lean-muscle mass (typical of athletes). Additionally, BMI measurements do not capture differences in body-fat distribution. Fat accumulation in the abdominal region, particularly of visceral fat, is predictive of multiple adverse health outcomes independent of overall BMI.^58^ We thus sought to validate our G × BMI findings by performing the same analysis on a subset of the TwinsUK cohort that had DXA-measured abdominal visceral fat volume. Within this subset (n = 682), visceral fat ranges from 78 to 1,542 g (mean = 627 g) and, as expected, broadly correlates with BMI (Figure S7). All 16 G × BMI interactions (FDR = 5%) showed a similar effect in the G × visceral fat analysis, and despite the drop in sample size, some interactions increased in significance by four orders of magnitude (*CAST* p = 3.0 × 10^−16^, *PEPD* p = 3.0 × 10^−15^) (Figure 3C), potentially due to the increased sensitivity gained from an accurate machine-measured phenotype. This provides evidence that G × BMI can capture real adiposity effects and suggests that, although more difficult to obtain, power to detect interactions increases dramatically with phenotypic measurement accuracy, such as in measurements obtained by DXA.

## Discussion

Here, we describe the pervasive effect BMI has on peripheral-tissue gene expression and identify robust examples of BMI-dependent regulatory variants. We characterize the properties of G × BMI regulatory variants, showing that they typically have strong main effects and are highly tissue specific. Additionally, we identify a G × BMI interaction that regulates an adipose-specific *trans*-network of 53 genes. Although twin and family studies estimate that ∼60% of expression heritability acts in *trans*,^23^, ^24^ *trans* effects have been difficult to identify in humans. Given the high context specificity of *trans* effects in model organisms,^10^ *trans* eQTL discovery efforts in humans might be more fruitful when accounting for environmental interactions.

These findings suggest that identifying G × E interactions on gene expression is possible with significantly smaller sample sizes, compared to complex traits. However, we note that the current study is underpowered, and we expect more G × BMI interactions to be identified with larger sample sizes. Extrapolating from empirical investigation of the power to detect cell-type-specific interactions in whole-blood transcriptomes,^20^ we expect that power to detect G × BMI effects on expression should scale linearly with sample size. Increasing sample size will most likely necessitate combining data across populations, which can confound G × E analysis if the environment differs across study populations. Using a physiological variable such as BMI should mitigate this caveat, however, we acknowledge that BMI could also be acting as a surrogate for a highly correlated cofactor, such as diet, which might vary across populations and thus complicate replication or meta-analysis efforts. Body-fat traits have been shown to be sexually dimorphic. It is therefore important to point out that although we demonstrated replicated examples of G × BMI effects in a separate adipose tissue cohort, the discovery sample was an all-female cohort, whereas 44% of the replication cohort were men, potentially reducing our replicative power.

It is well documented that increasing BMI induces changes in the cell-type composition and inflammation of adipose tissue.^59^, ^60^, ^61^, ^62^, ^63^ Changes in cell-type composition could be the underlying mechanism of some of our identified G × BMI interactions. This is an intriguing possibility, and we are actively investigating methods to deconvolve cell-type expression data from whole-tissue expression profiles to address this. Enrichment for G × BMI genes in immune response pathways could also represent a change in the activation state of cells already present in adipose tissue or in the genetic control of inflammation susceptibility that takes place under weight gain, during which some individuals, due to their genotype, could undergo less inflammation in an obese state. Given the enrichment for genes involved in metabolic processes, other potential mechanism include BMI-driven changes in metabolism,^64^ adipocyte size^65^ (a consequence of having to store more lipid droplets in an obese state),^66^ increased vascularization as a result of hypoxia,^67^ and changes in overall energy expenditure.^68^

Discovery of G × BMI effects on gene expression has broad applicability. Characterization of G × BMI regulatory variants and the genes they act upon will help elucidate the downstream consequences of obesity and the molecular pathways leading to associated diseases. Similar to the widespread use of main-effect eQTLs to interpret GWAS loci, context-specific eQTLs can be used both to identify the molecular mechanism of GWAS loci and to identify relevant, interacting environments or risk factors. To facilitate this, we have made the full results of this study freely available in an interactive web service. Finally, identifying individuals whose genotypes predispose them to BMI-specific outcomes could enable targeted interventions for those most likely to respond and could improve accuracy in assessing genotypic risk of obesity-related diseases.

## Figures and Tables

**Table 1:** Significant *cis* G × BMI Regulatory Interactions in Adipose, FDR = 5%

**Gene**	**SNP**	**EA**	**EAF**	**TwinsUK β**	**TwinsUK p Value**	**deCODE β**	**deCODE p Value**	**Main-effect eQTL (FDR = 1%)**	**Enhancer**
*CHURC1*	rs7143432	A	0.78	0.026	2.0 × 10^−12^	0.011	8.5 × 10^−4^	adipose, skin, blood, LCLs	–
*CAST*	rs13160562	G	0.69	−0.032	3.9 × 10^−12^	−0.004	0.053^a^	adipose, skin, blood, LCLs	adipocyte
*CIDEA*	rs7505859	C	0.62	−0.028	3.1 × 10^−11^	−0.004	0.21	adipose, skin	adipocyte
*ZNF117*	rs6948760	T	0.40	0.039	4.4 × 10^−11^	0.0009	0.79	adipose, skin, blood, LCLs	–
*ADH1A*	rs1693457	C	0.18	0.034	5.9 × 10^−11^	−0.0015	0.85^a^	adipose	adipocyte
*RP11-71E19.1*	rs1980140	A	0.79	−0.058	6.1 × 10^−11^	NA	NA	adipose	adipocyte
*PEPD*	rs10415555	A	0.81	−0.044	4.8 × 10^−10^	−0.014	4.2 × 10^−6^	adipose, skin	adipocyte
*ANXA5*	rs2306420	G	0.71	0.022	1.4 × 10^−9^	0.001	0.52	adipose, skin, blood, LCLs	adipocyte
*SIK1*	rs12482956	A	0.71	0.058	3.0 × 10^−9^	NA	NA	–	blood
*HLA-DQB2*	rs114370295	T	0.27	−0.050	3.5 × 10^−9^	−0.004	0.45	adipose	–
*ERV3-1*	rs11979998	C	0.52	0.032	8.4 × 10^−9^	−0.0008	0.84^a^	adipose, skin, blood, LCLs	blood
*POU6F2*	rs34792397	G	0.75	−0.041	9.9 × 10^−9^	NA	NA	adipose	–
*IFNAR1*	rs2834098	C	0.78	−0.047	1.4 × 10^−8^	0.002	0.58	–	stem cells
*SCFD2*	rs7687982	A	0.75	−0.059	1.5 × 10^−8^	−0.006	0.26	–	aMSC
*PHACTR3*	rs6070866	G	0.51	−0.044	1.7 × 10^−8^	−0.020	1.1 × 10^−4^	adipose	brain
*SPAG17*	rs9661038	G	0.64	0.043	2.8 × 10^−8^	−0.004	0.083^a^	–	–

Exact exon coordinates are listed in Tables S1 and S3. The main-effect column lists the TwinsUK tissues where the listed gene-SNP pair has a main-effect *cis* eQTL when tested without the interaction effect (a dash denotes no eQTL in any tissue). All interaction effects were adipose specific. If a SNP or its proxy (r^2^ > 0.8) falls within an enhancer as defined by ENCODE, the cell type that enhancer is primarily active in is listed in the Enhancer column. EA, effect allele; EAF, effect-allele frequency in the TwinsUK discovery sample.

a deCODE exons with less than 90% overlap with the corresponding deCODE exon (see methods, Table S1).

## Accession Numbers

The accession number for the RNA-seq data reported in the paper is EGA: EGAS00001000805.

## Web Resources

European Genome-phenome Archive (EGA), https://www.ebi.ac.uk/ega
OMIM, http://www.omim.org/
PhenoExpress, BMI transciptome-wide association study and G × BMI visualization server, http://expression.kcl.ac.uk
RefSeq, http://www.ncbi.nlm.nih.gov/RefSeq
UCSC Genome Browser, http://genome.ucsc.edu

## Supplemental Data

Document S1. Figures S1–S7 and Tables S1, S2, S4, and S8 Document S1. Figures S1–S7 and Tables S1, S2, S4, and S8

Document S2. Tables S3 and S5–S7 Document S2. Tables S3 and S5–S7

Document S3. Article plus Supplemental Data Document S3. Article plus Supplemental Data
