# Supplementary material for: Adiposity-Dependent Regulatory Effects on Multi-tissue Transcriptomes
Source: Am J Hum Genet. 2016 Sep 1;99(3):567–79. doi: 10.1016/j.ajhg.2016.07.001 (PMC5011064; doi:10.1016/j.ajhg.2016.07.001)
Supplement: Document S1. Figures S1–S7 and Tables S1, S2, S4, and S8 [file mmc1.pdf]

The American Journal of Human Genetics, Volume *99*

Supplemental Data

Adiposity-Dependent Regulatory Effects

on Multi-tissue Transcriptomes

Craig A. Glastonbury, Ana Viñuela, Alfonso Buil, Gisli H. Halldorsson, Gudmar Thorleifsson, Hannes Helgason, Unnur Thorsteinsdottir, Kari Stefansson, Emmanouil T. Dermitzakis, Tim D. Spector, and Kerrin S. Small

Supplemental materials

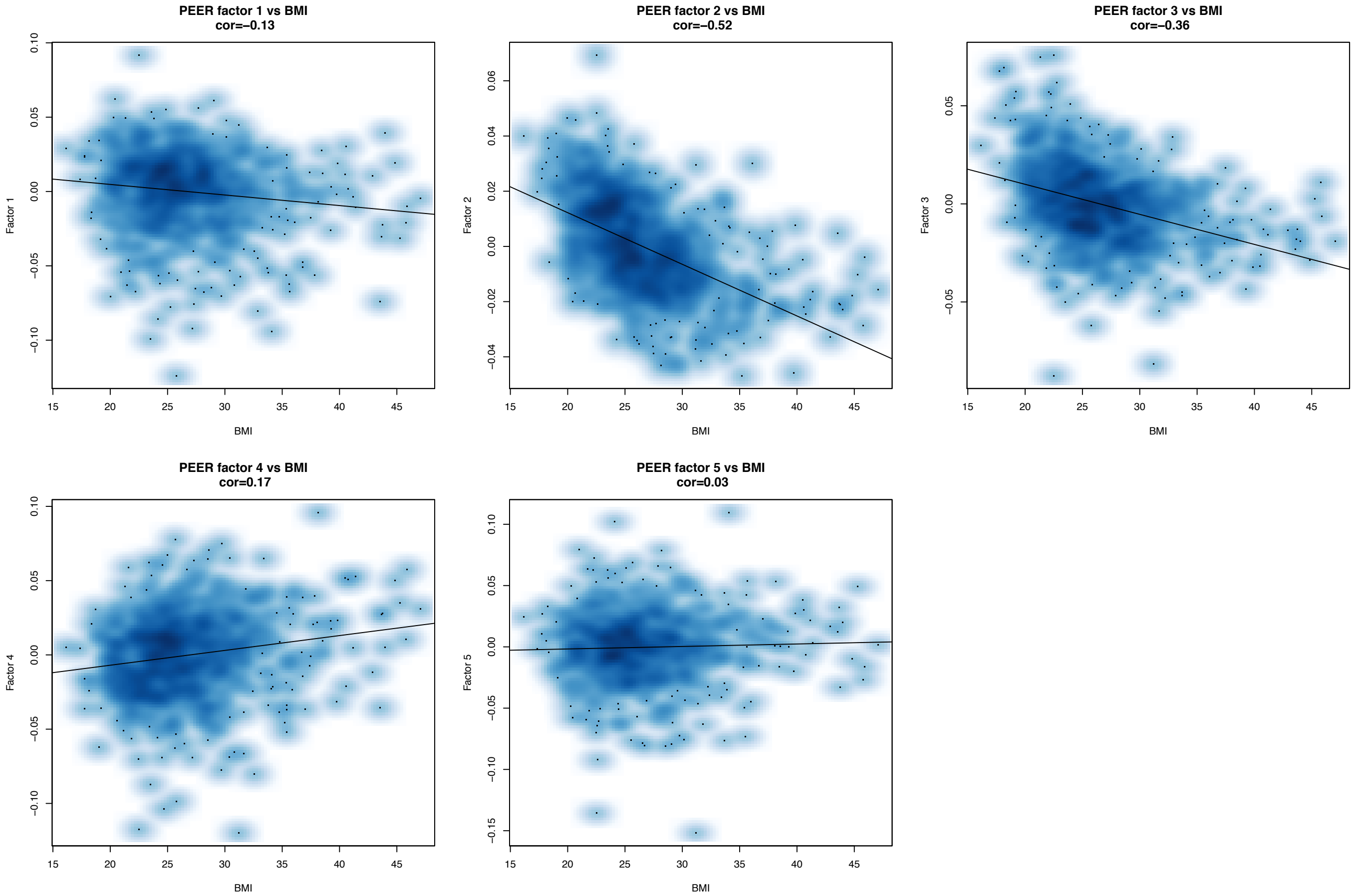

Figure S1: Correlation between first five PEER factors and BMI, our environment of interest. Removing latent factors increases our ability to find genetic effects that interact with our environment of interest by reducing model co-linearity and accounting for latent hidden factors.

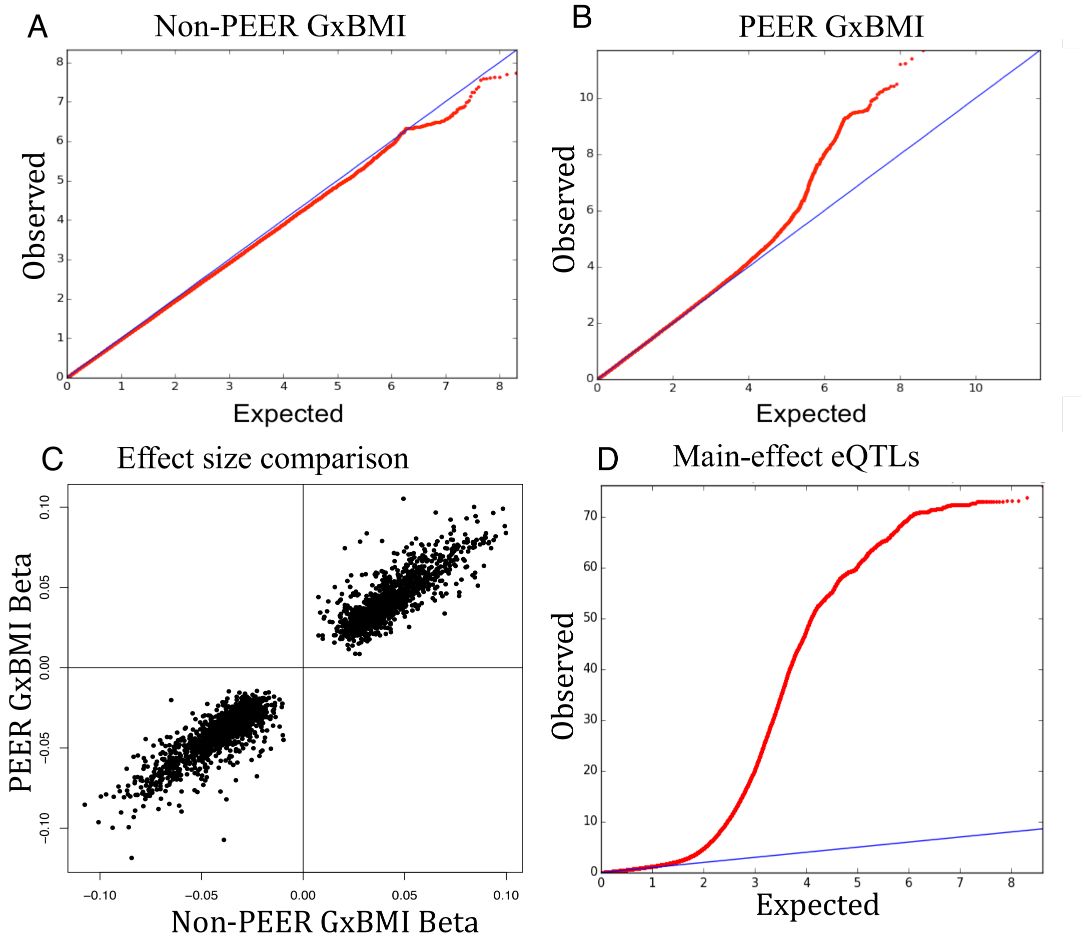

Figure S2: A) QQ-plot of the non-PEER GxBMI interaction discovery *P*-values compared to a standard uniform distribution (expected *P*-values). B) QQ-plot after PEER correction. C) Comparison of interaction effect estimates using non-PEER and PEER expression residuals. D) QQ-plot of standard main effect eQTL *P*-values, recapitulating the previously well established enrichment of eQTLs (Cusanovitch *et al.,* 2016).

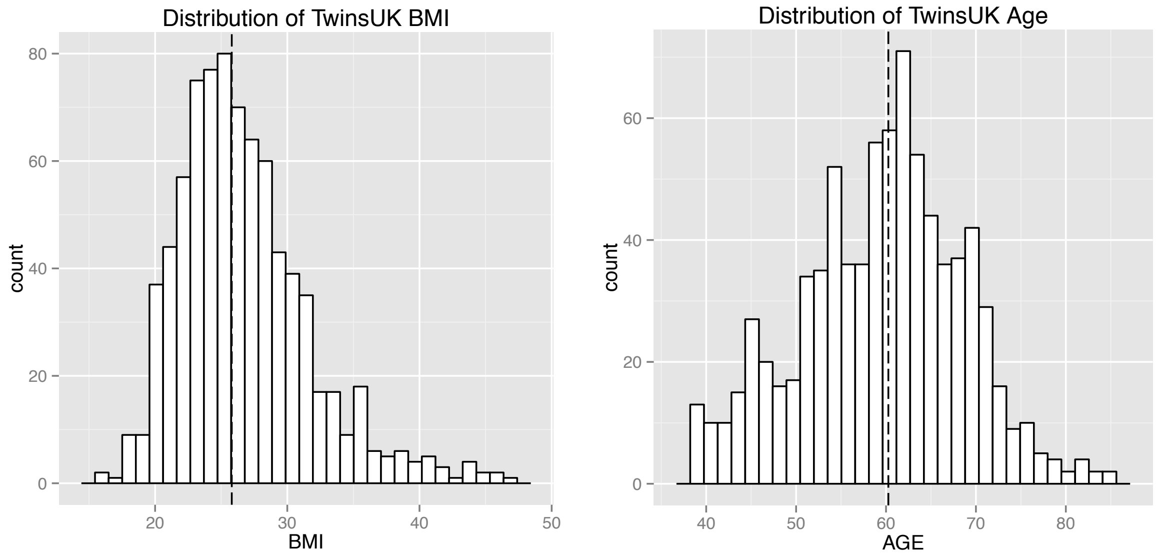

Figure S3: BMI and age distribution of twins present in this study. Dashed line represents median BMI and age.

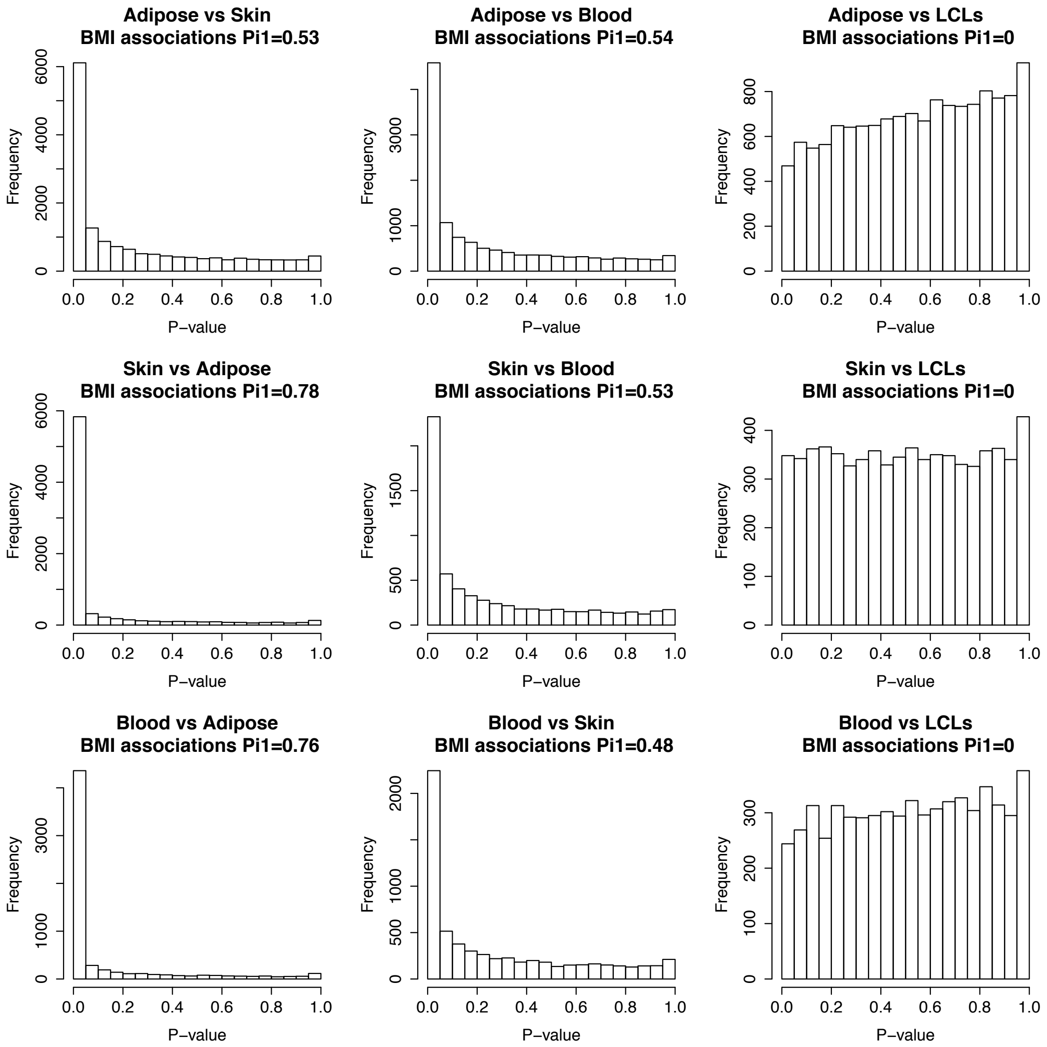

Figure S4: Tissue-specificity of association between BMI and expression. By taking the exons associated with BMI in one tissue and intersecting the results of BMI associations in another tissue, we can assess tissue specificity/sharing. For example, the upper left histogram shows the p value distribution of the association between BMI and skin expression of all exons found to be associated to BMI in adipose tissue. A π_1 value of 0.53 indicates approximately half of the BMI associations in adipose are found in skin

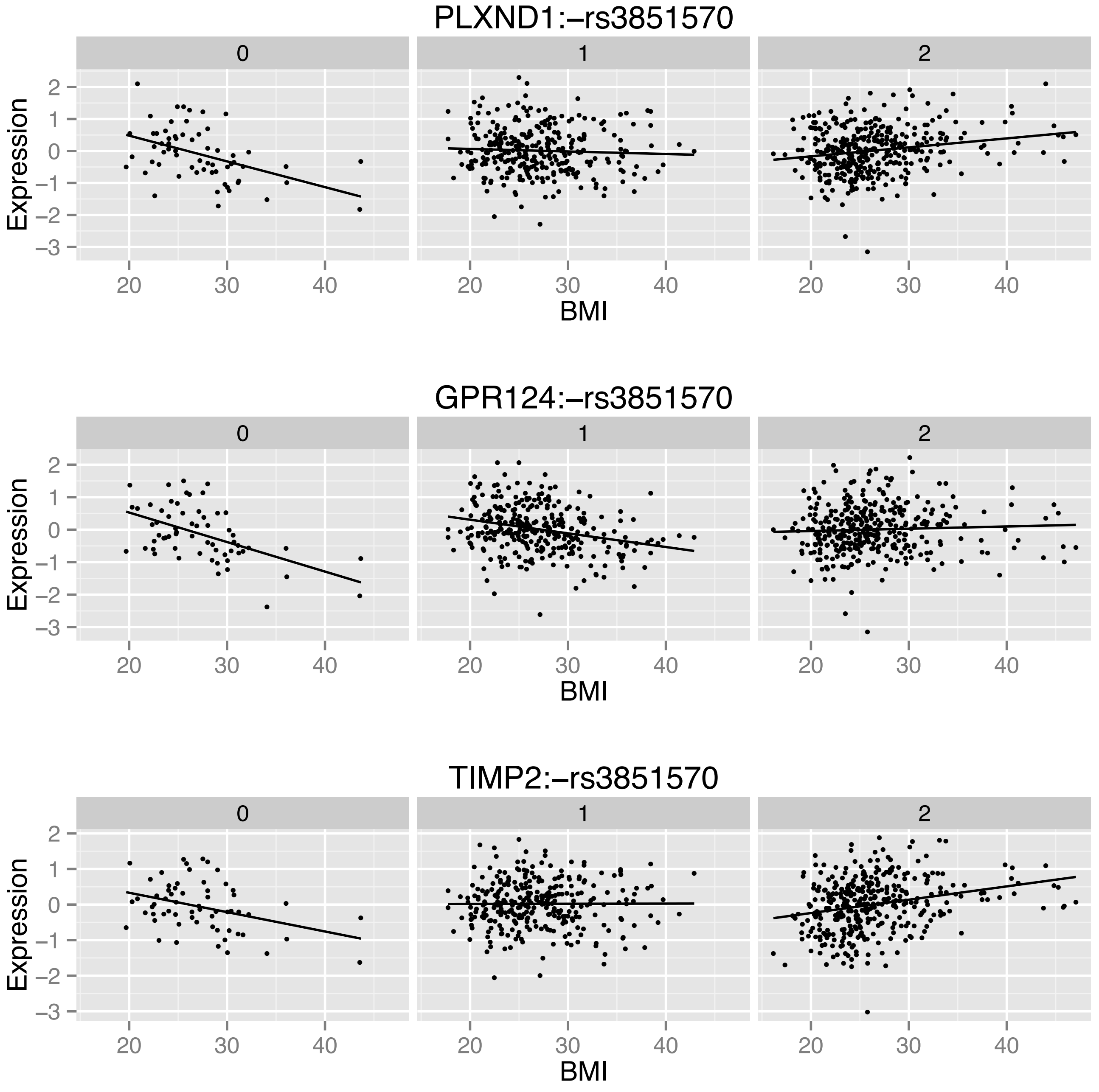
PLXND1:−rs3851570
GPR124:−rs3851570
TIMP2:−rs3851570
0
1
2
Expression
BMI
2
1
0
−1
−2
−3
20
30
40

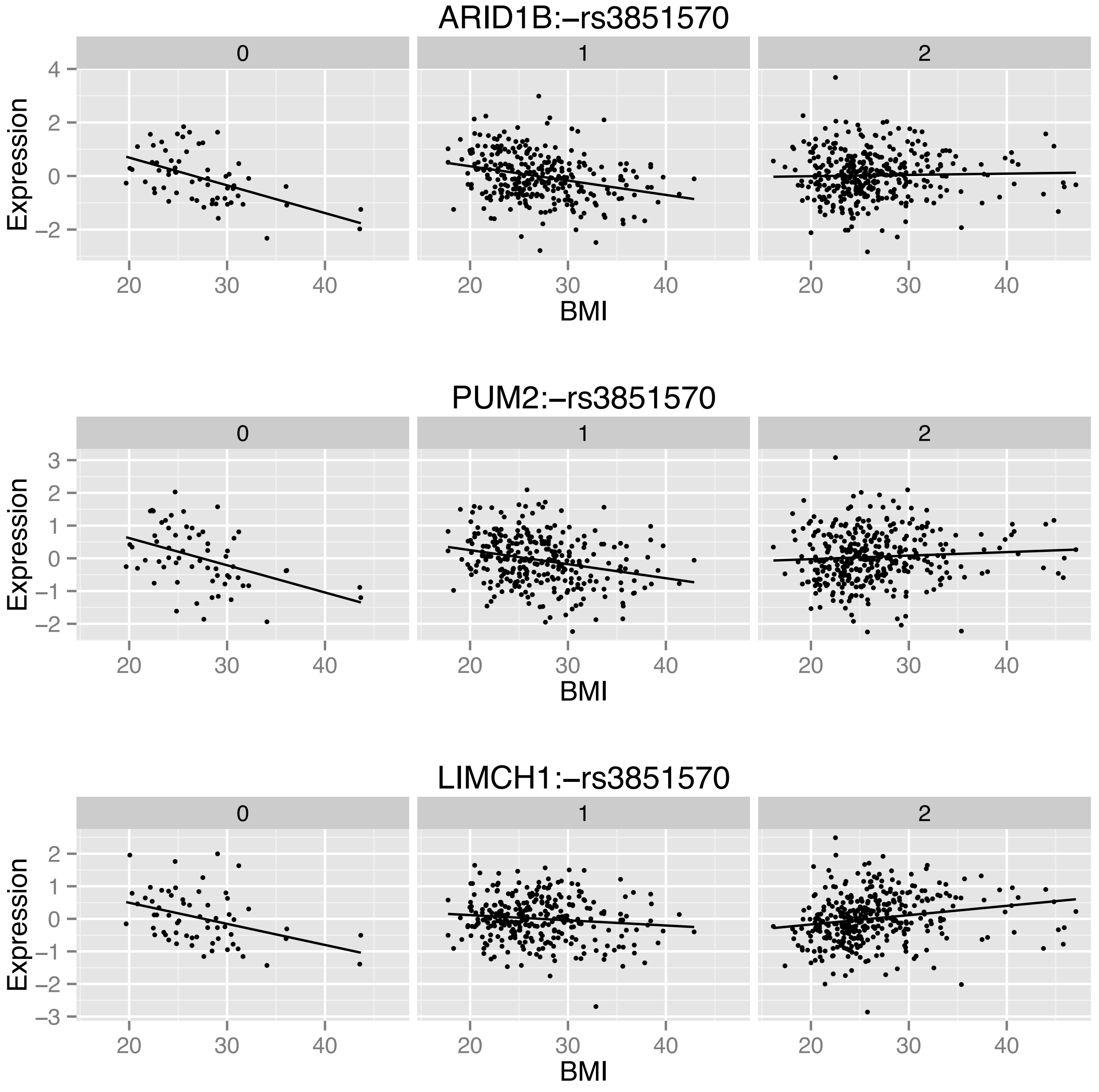

ARID1B:−rs3851570
PUM2:−rs3851570
LIMCH1:−rs3851570
0
1
2
Expression
BMI
20
30
40

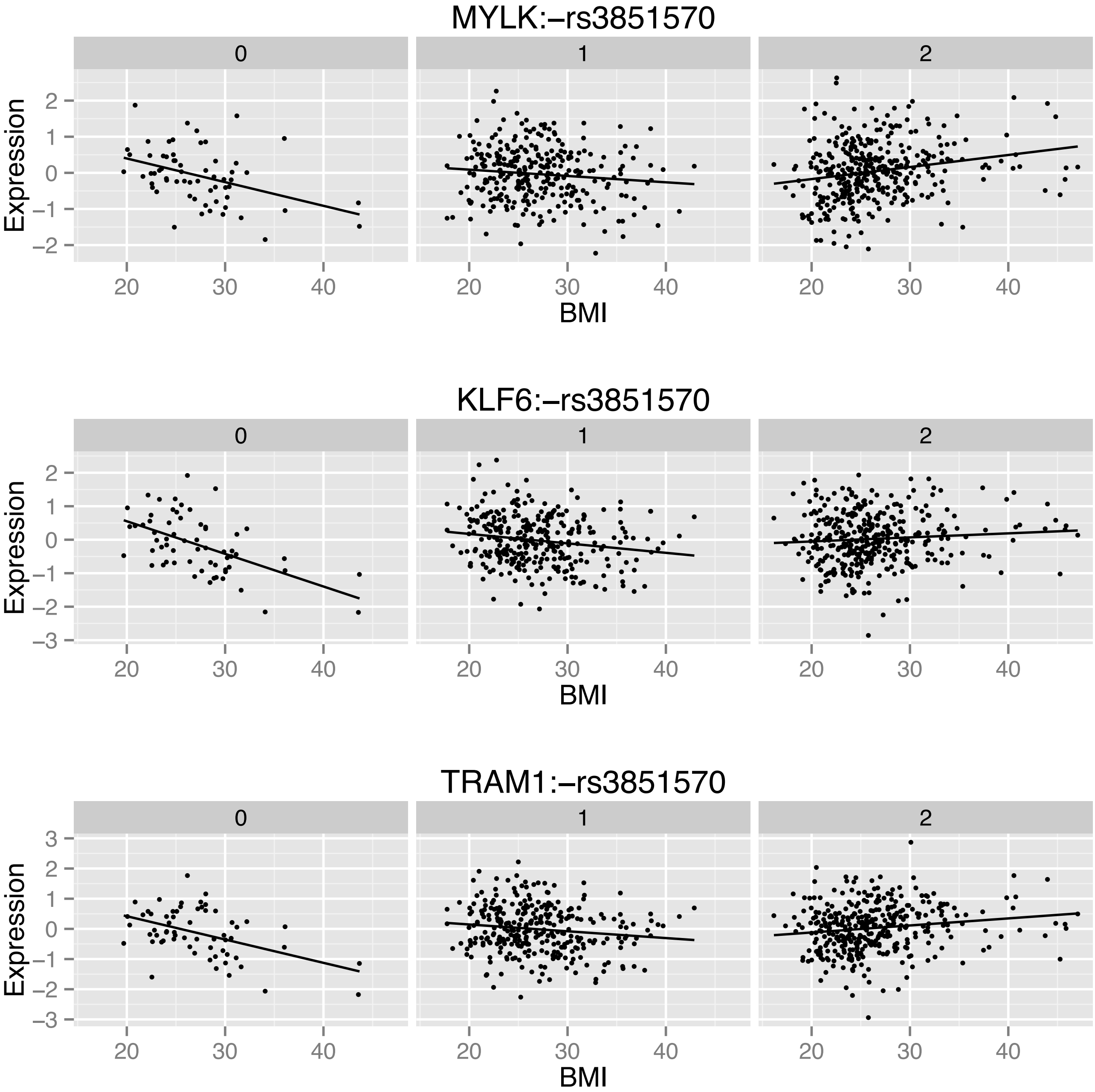
MYLK:−rs3851570
0
1
2
Expression
BMI
KLF6:−rs3851570
0
1
2
Expression
BMI
TRAM1:−rs3851570
0
1
2
Expression
BMI

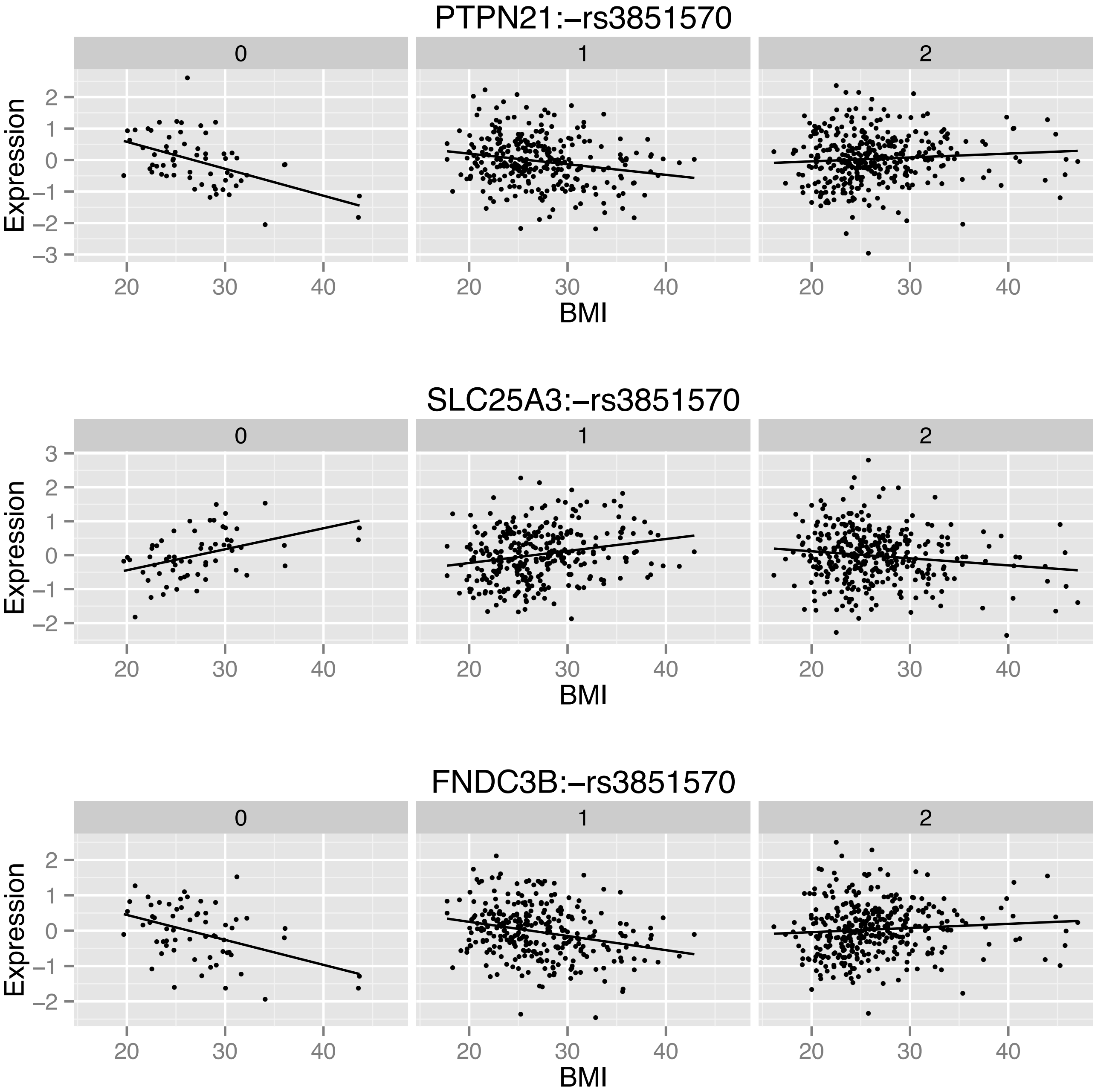

PTPN21:-rs3851570
SLC25A3:-rs3851570
FNDC3B:-rs3851570
0
1
2
Expression
BMI

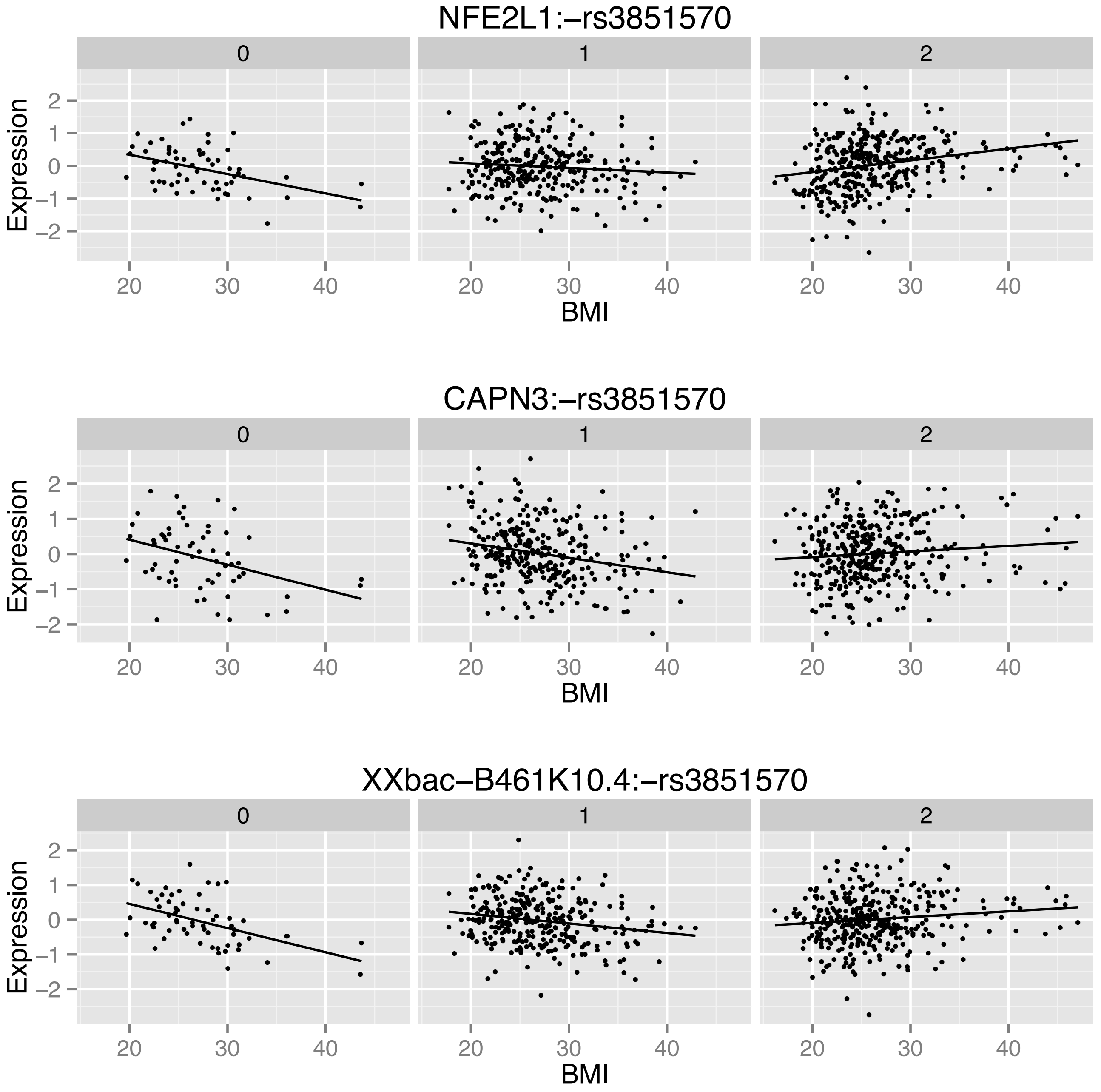

NFE2L1:–rs3851570
0
1
2
Expression
BMI
CAPN3:–rs3851570
0
1
2
Expression
BMI
XXbac–B461K10.4:–rs3851570
0
1
2
Expression
BMI

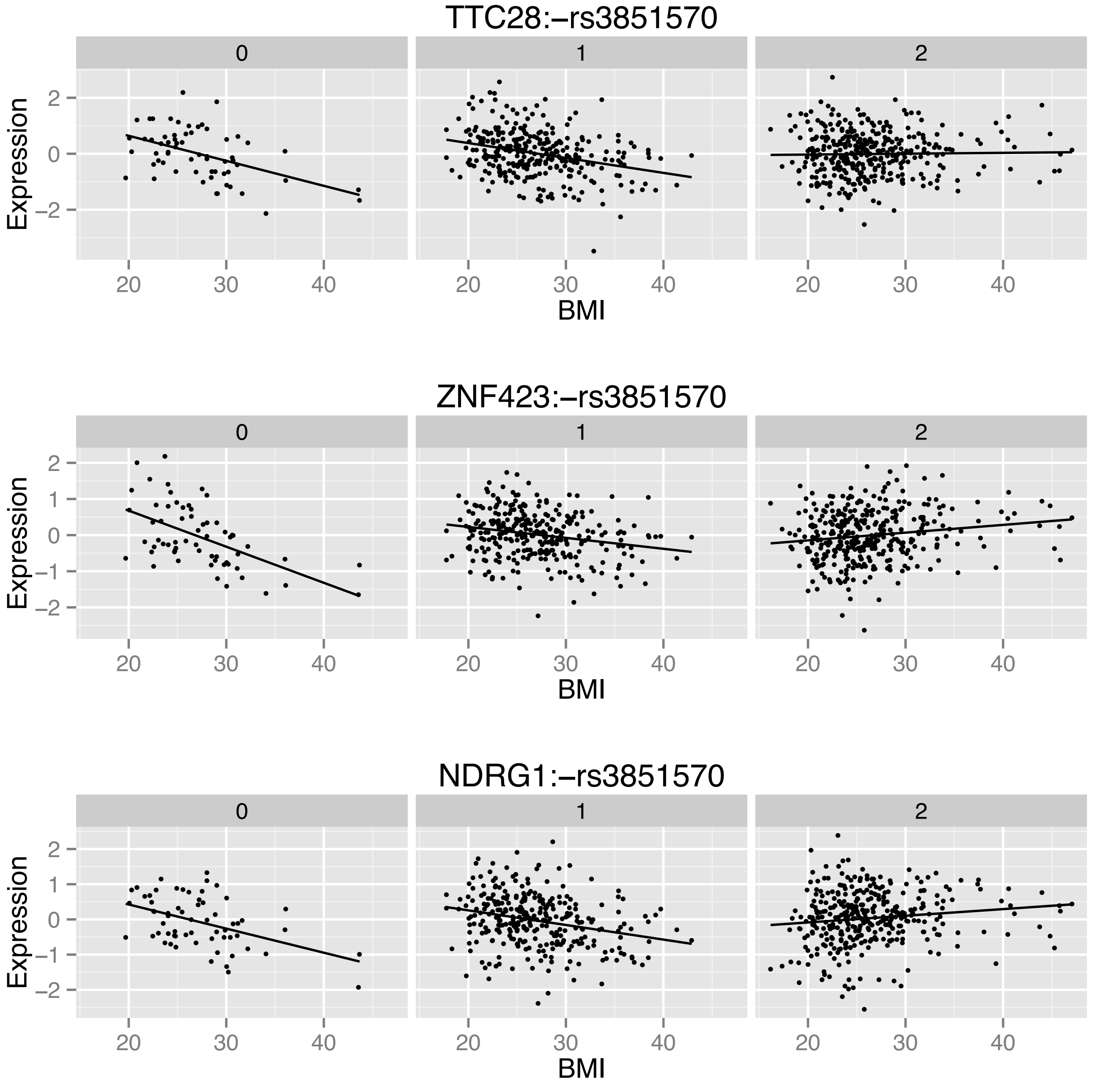
TTC28:-rs3851570
ZNF423:-rs3851570
NDRG1:-rs3851570
0
1
2
Expression
BMI
20
30
40
2
1
0
-1
-2

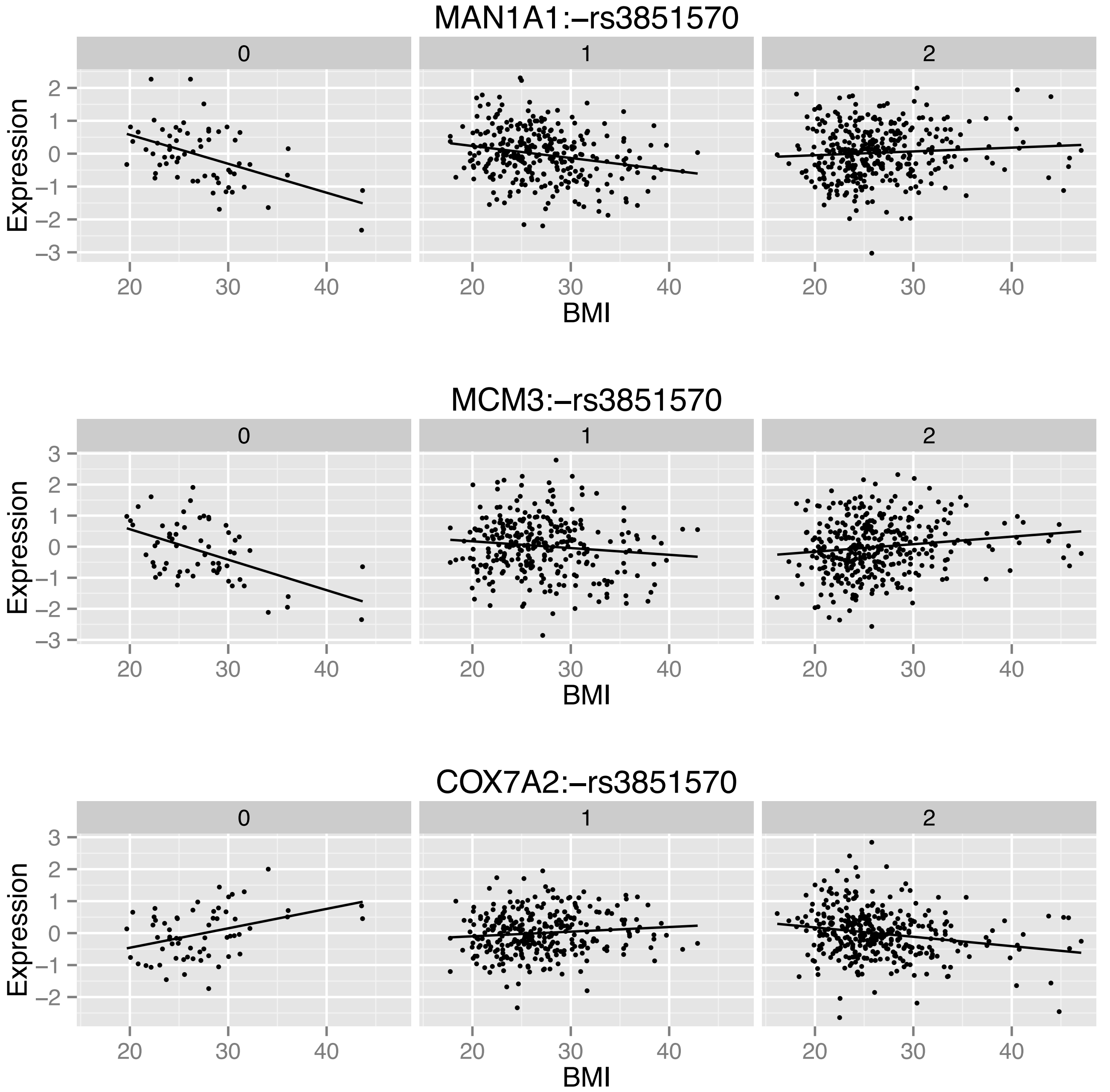

MAN1A1:-rs3851570
MCM3:-rs3851570
COX7A2:-rs3851570
0
1
2
Expression
BMI
20
30
40

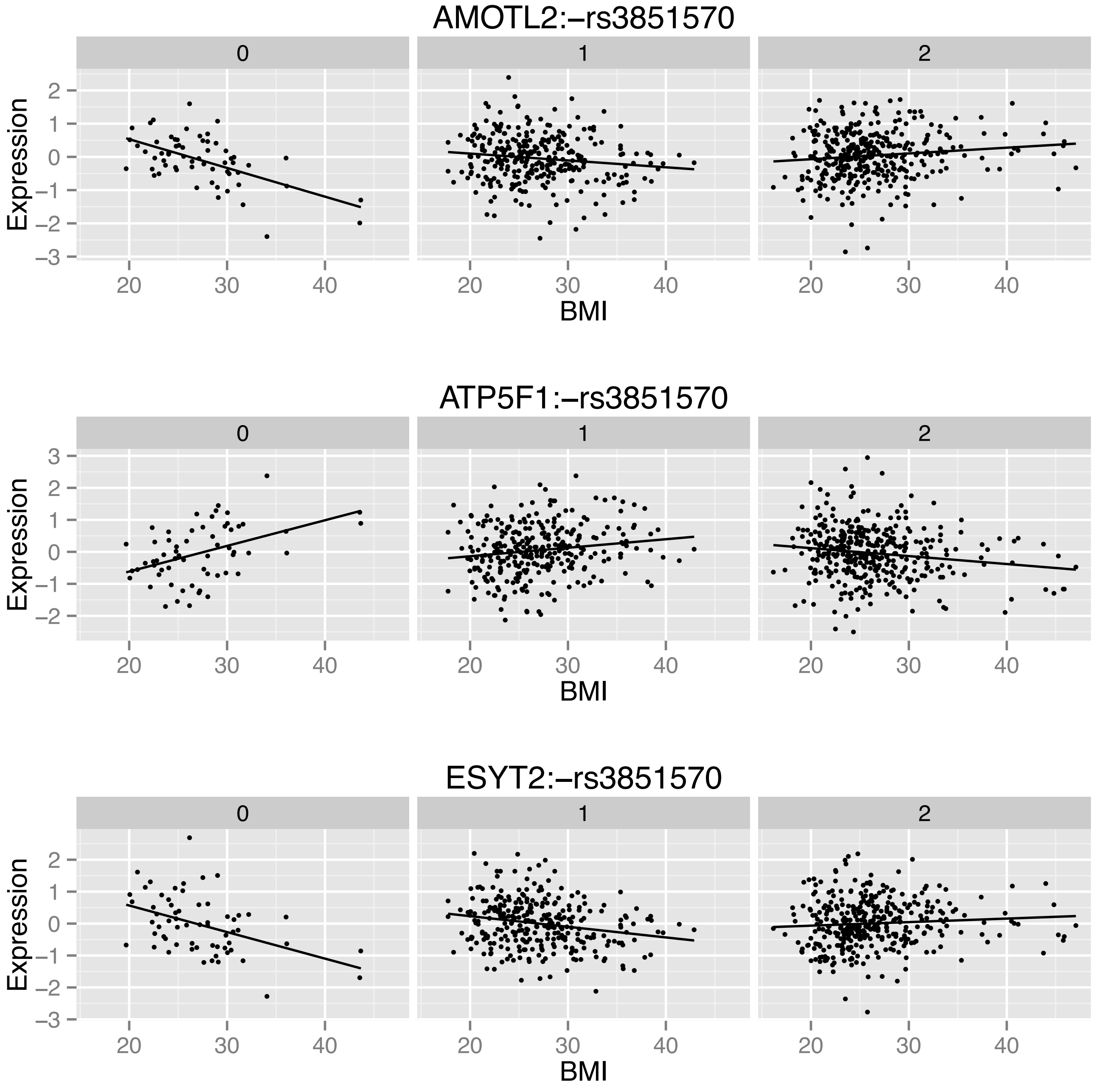
AMOTL2:–rs3851570
0
1
2
Expression
BMI
ATP5F1:–rs3851570
0
1
2
Expression
BMI
ESYT2:–rs3851570
0
1
2
Expression
BMI

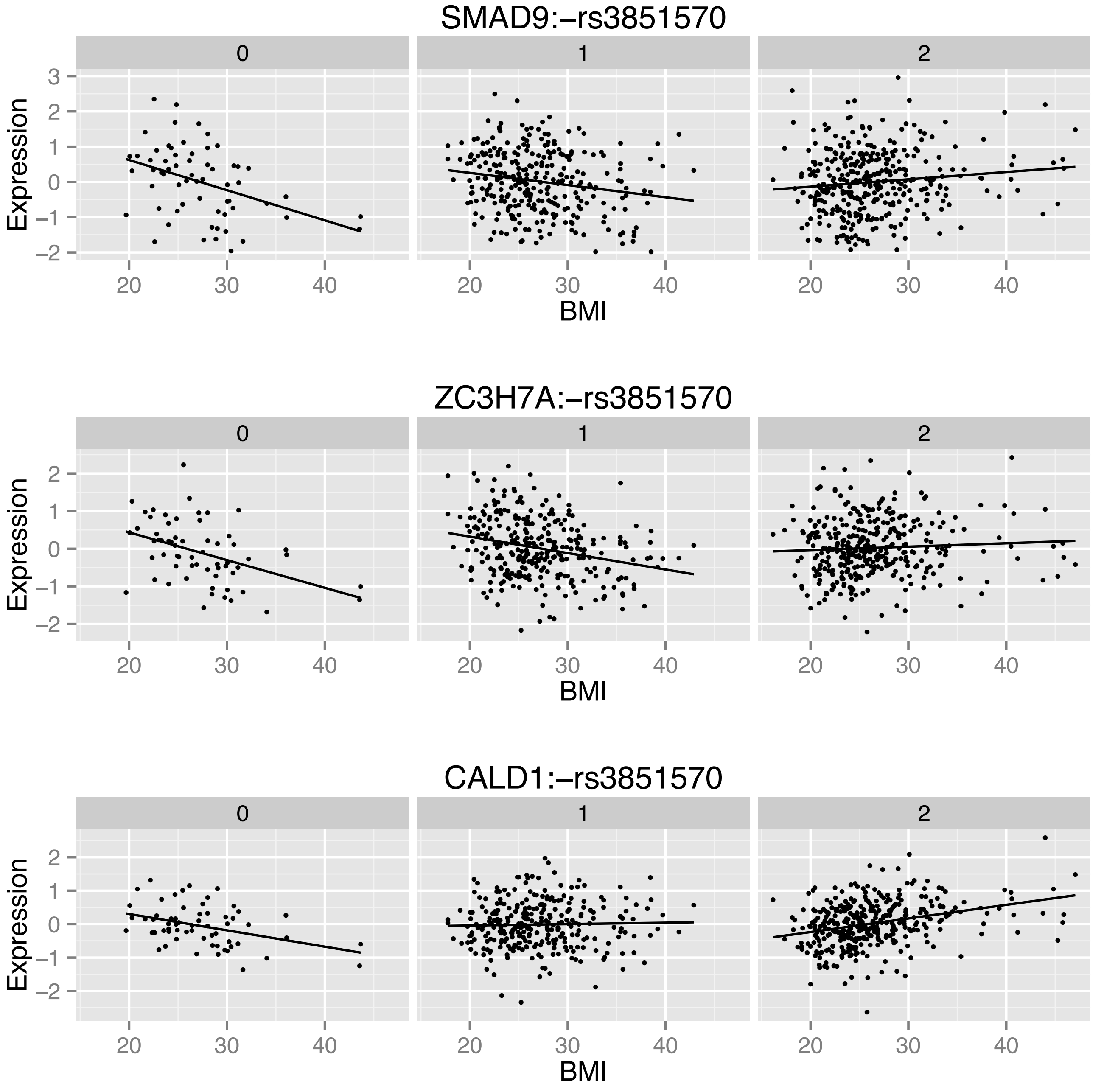
SMAD9:−rs3851570
0
1
2
Expression
BMI
ZC3H7A:−rs3851570
0
1
2
Expression
BMI
CALD1:−rs3851570
0
1
2
Expression
BMI

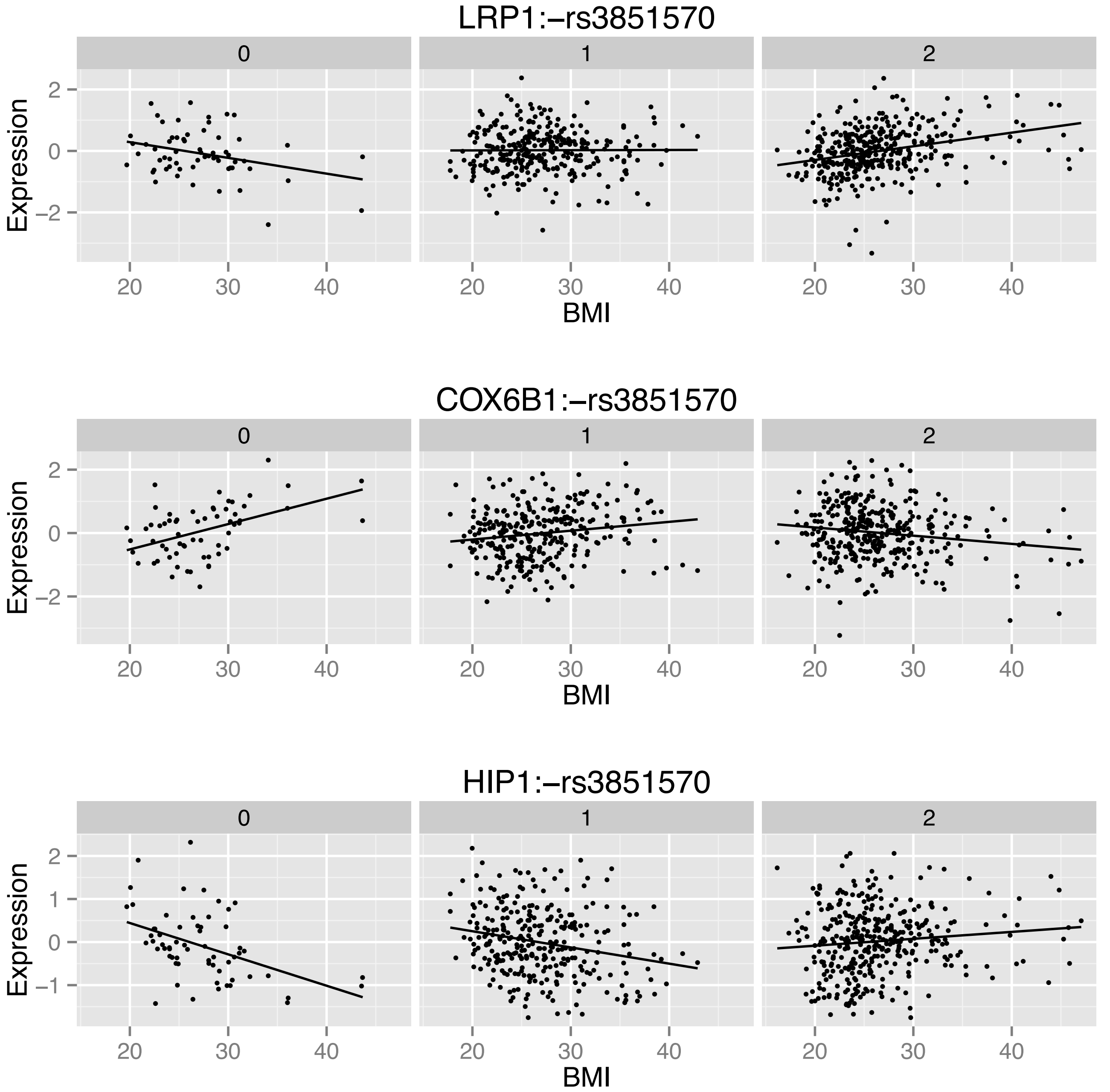
LRP1:−rs3851570
0
1
2
Expression
BMI
COX6B1:−rs3851570
0
1
2
Expression
BMI
HIP1:−rs3851570
0
1
2
Expression
BMI

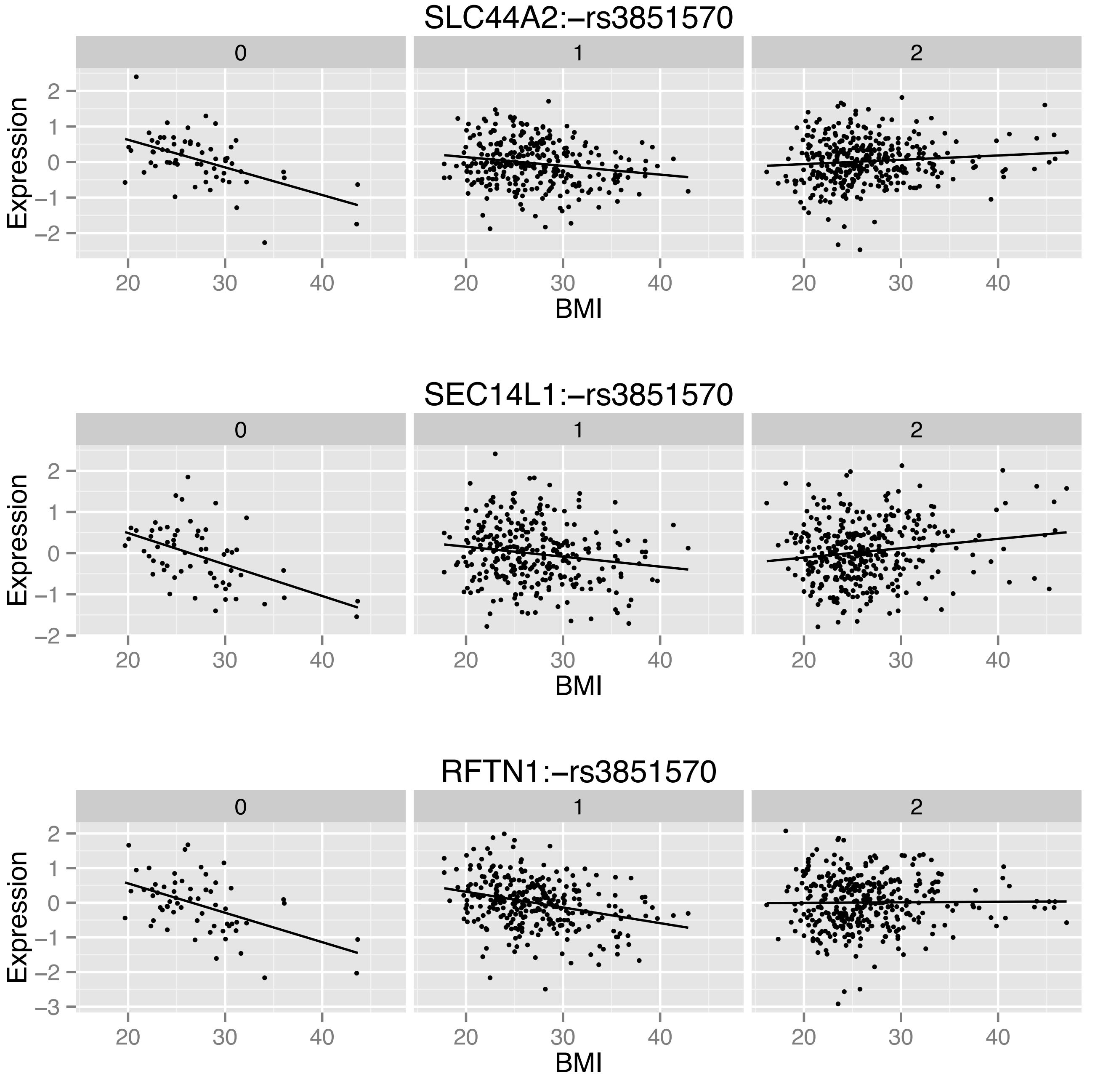
SLC44A2:–rs3851570
SEC14L1:–rs3851570
RFTN1:–rs3851570
0
1
2
Expression
BMI
20
30
40
2
1
0
−1
−2
−3

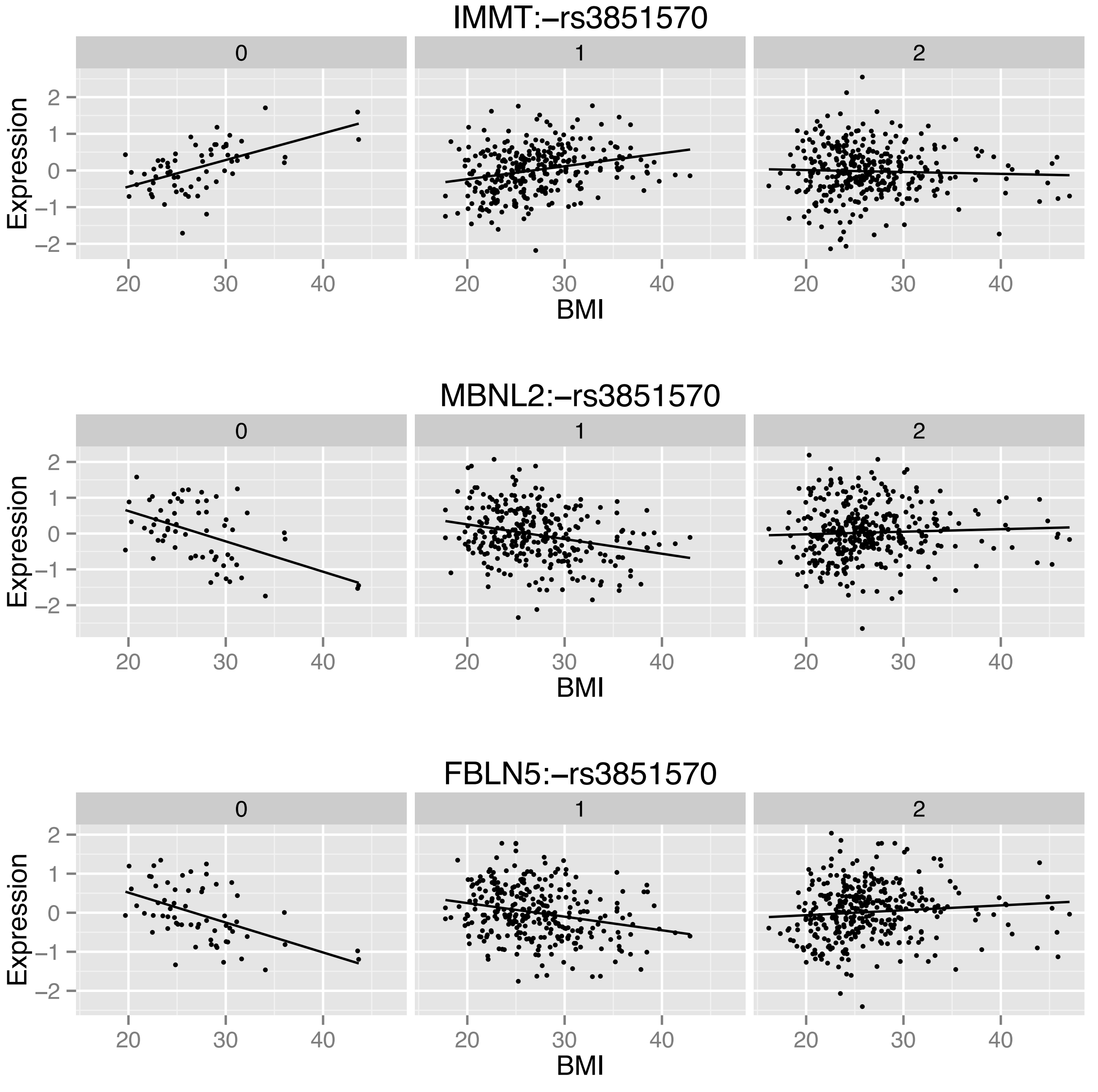
IMMT:–rs3851570
MBNL2:–rs3851570
FBLN5:–rs3851570
0
1
2
Expression
BMI
2
1
0
−1
−2
20
30
40

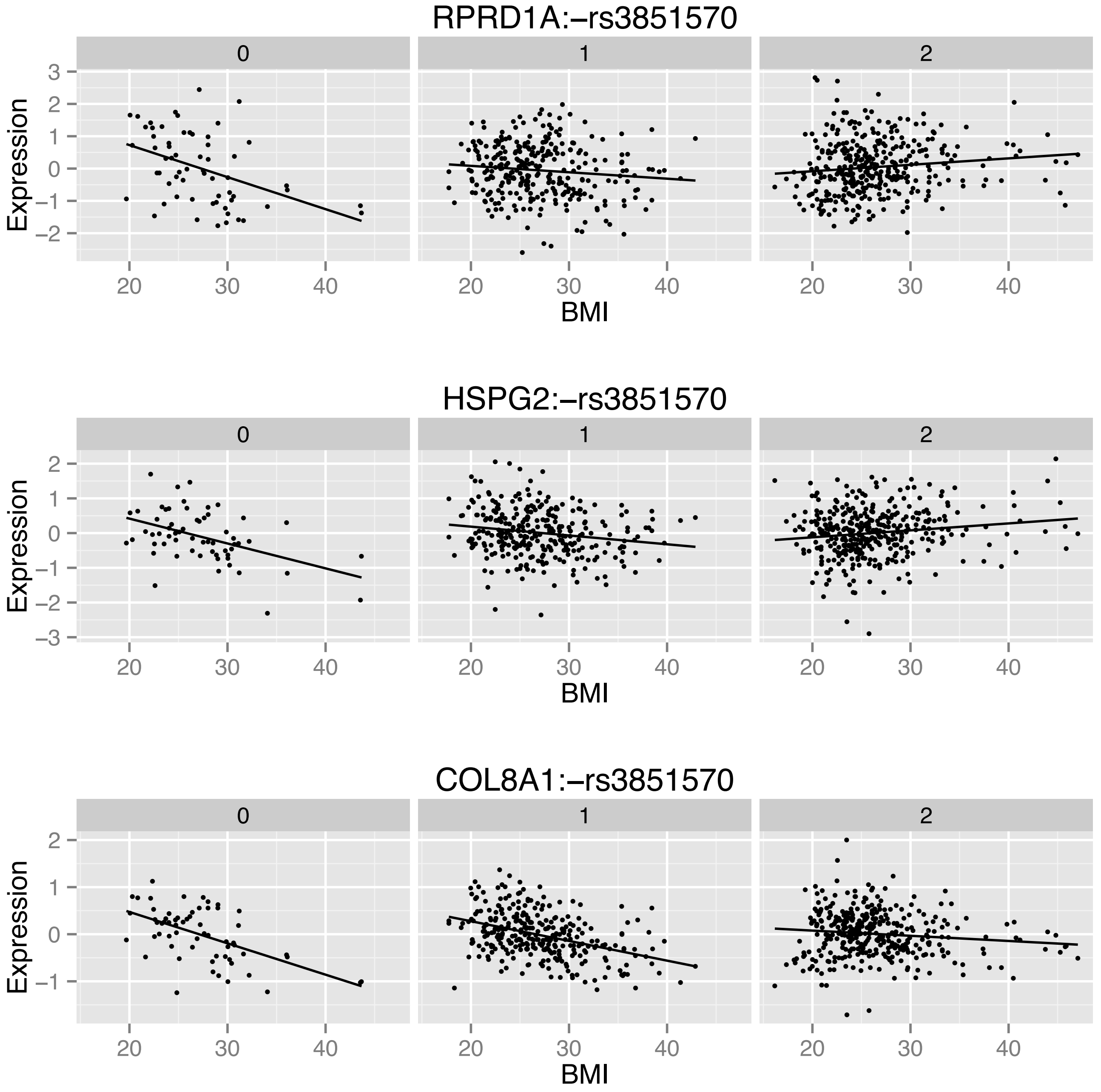

RPRD1A:-rs3851570
HSPG2:-rs3851570
COL8A1:-rs3851570
0
1
2
Expression
BMI
20
30
40

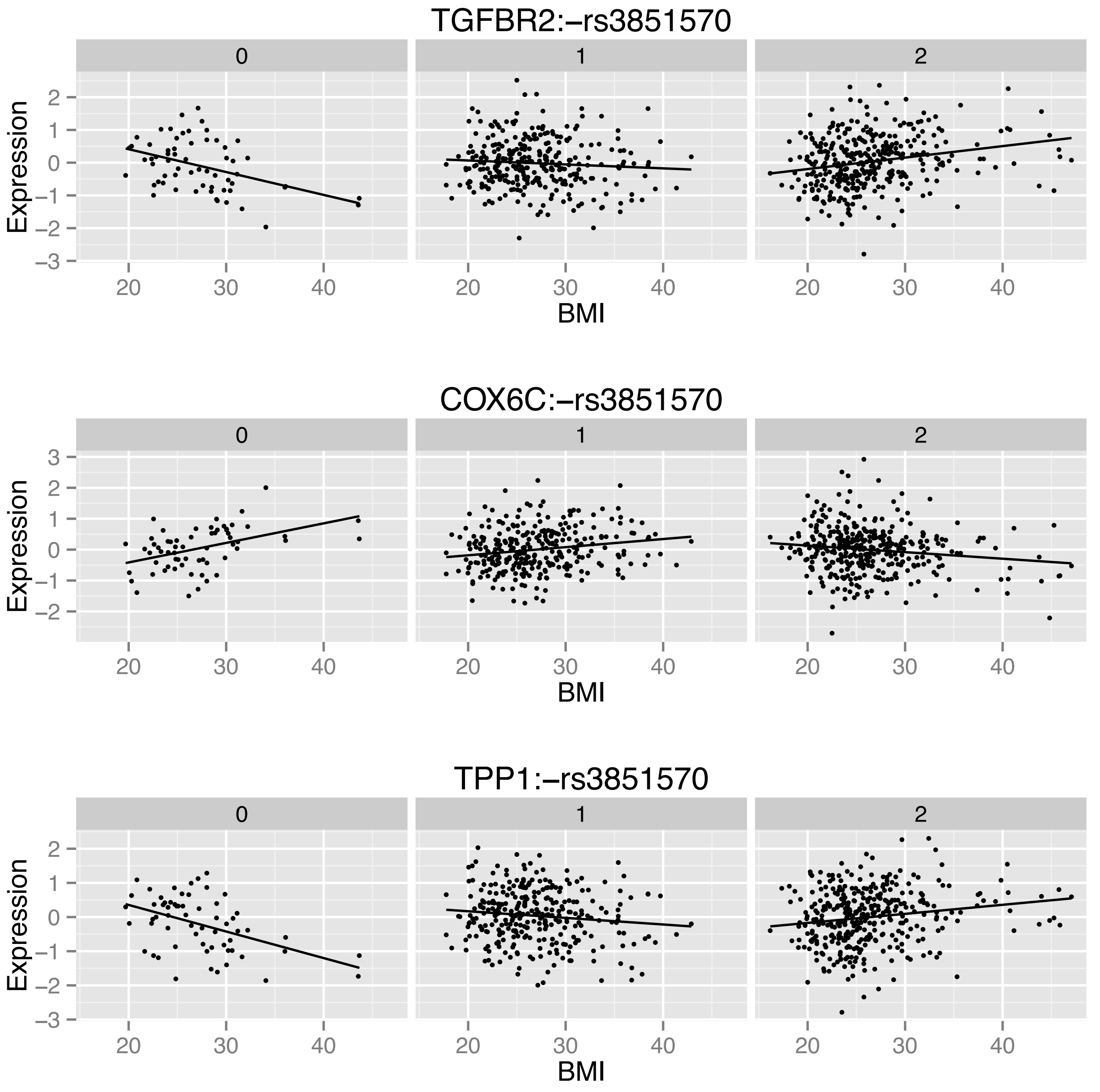
TGFBR2:–rs3851570
COX6C:–rs3851570
TPP1:–rs3851570
0
1
2
Expression
BMI
20
30
40

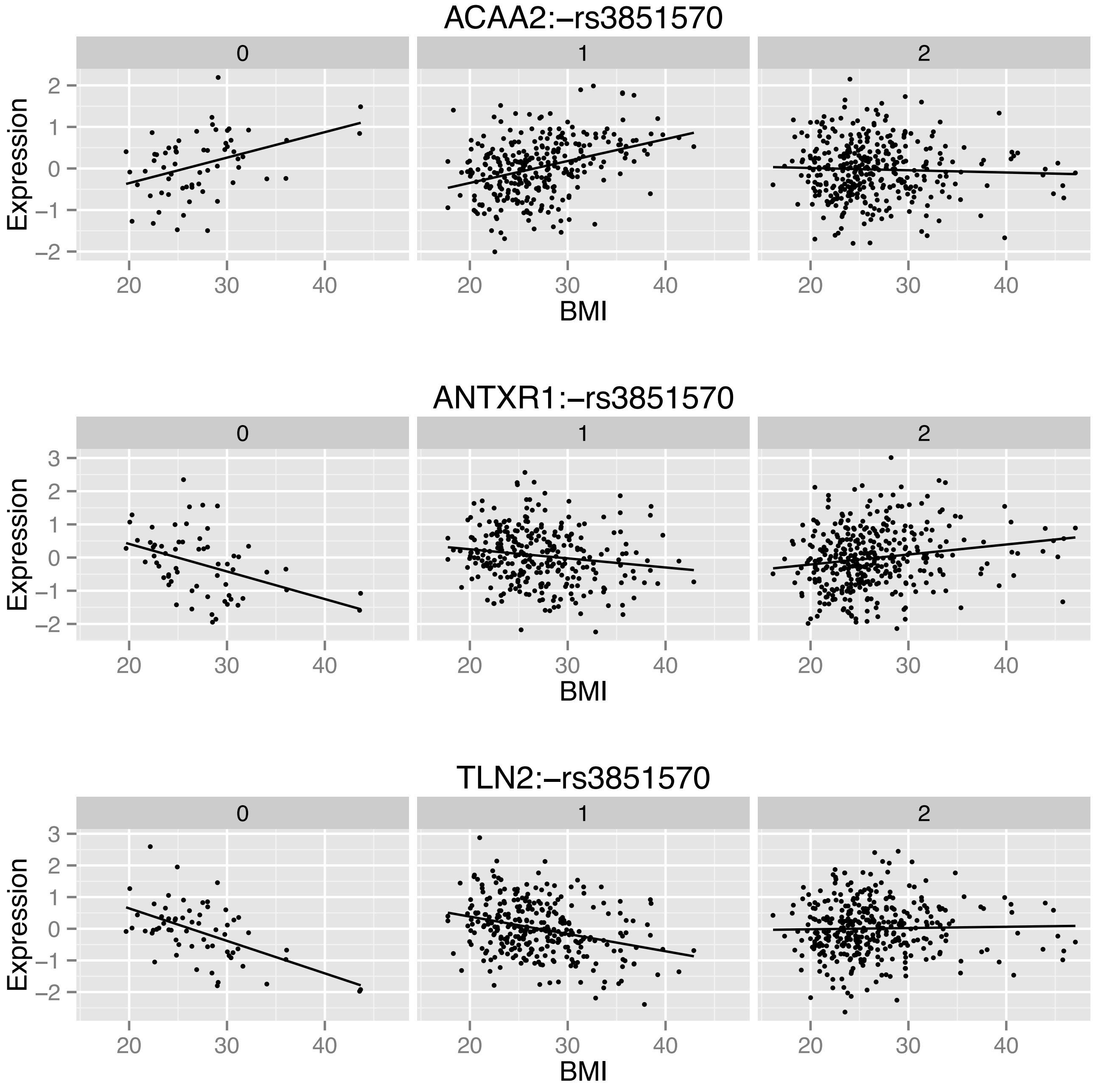
ACAA2:−rs3851570
ANTXR1:−rs3851570
TLN2:−rs3851570
0
1
2
Expression
BMI
20
30
40

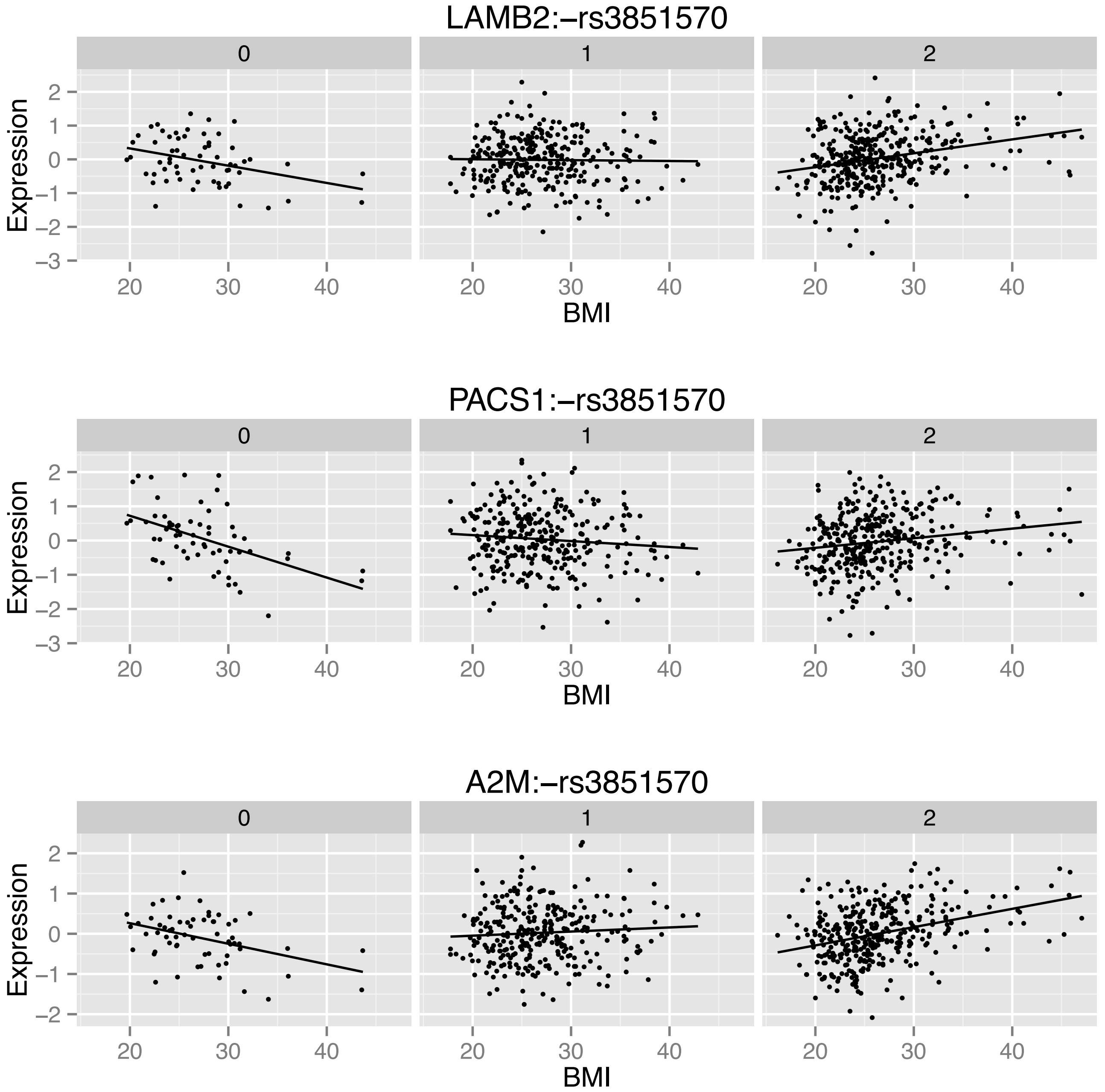

LAMB2:-rs3851570
PACS1:-rs3851570
A2M:-rs3851570
0
1
2
Expression
BMI
20
30
40
2
1
0
-1
-2
-3

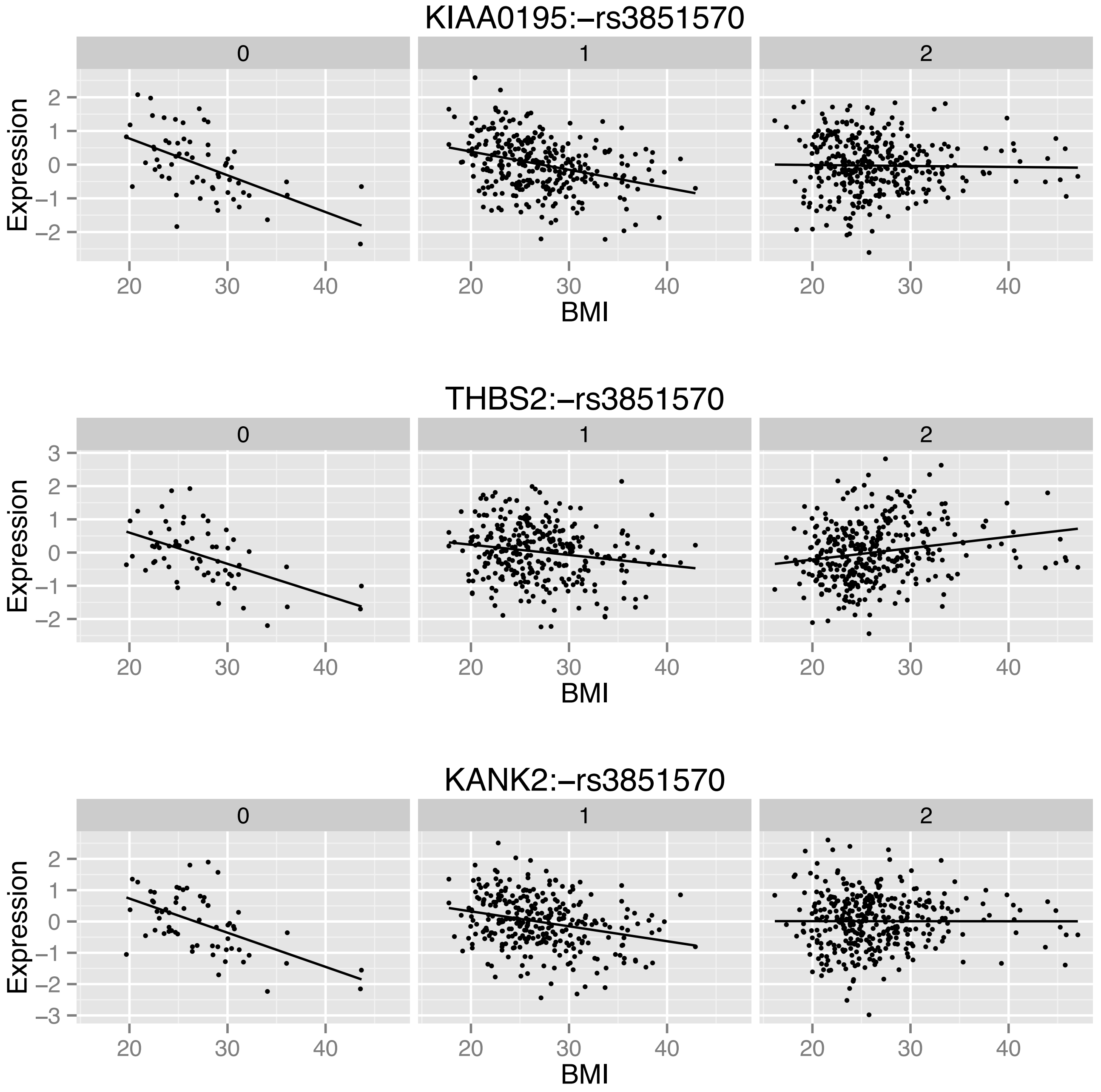
KIAA0195:−rs3851570
THBS2:−rs3851570
KANK2:−rs3851570
0
1
2
Expression
BMI
20
30
40

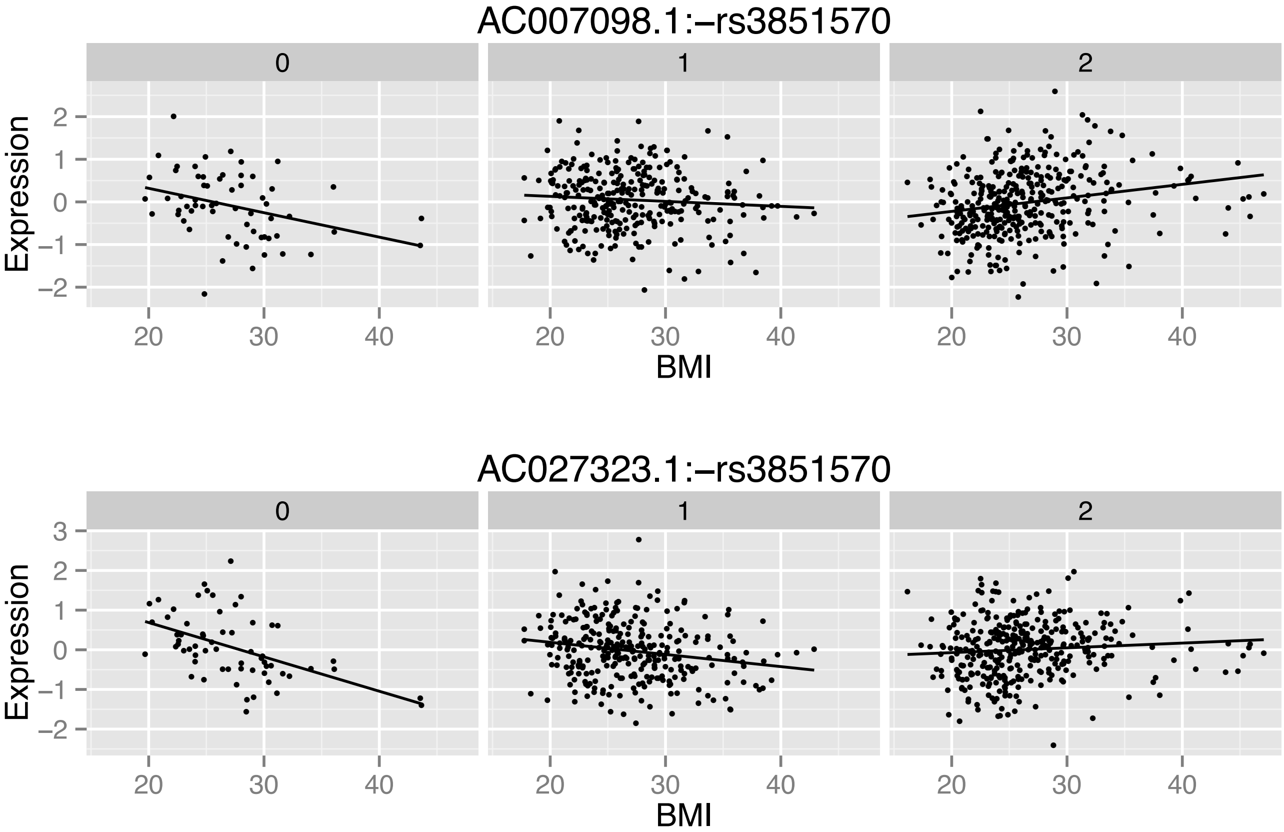

Figure S5: All 53 trans GxBMI associations

Adipose GxBMI SNP-gene pair matched to eQTL datasets:

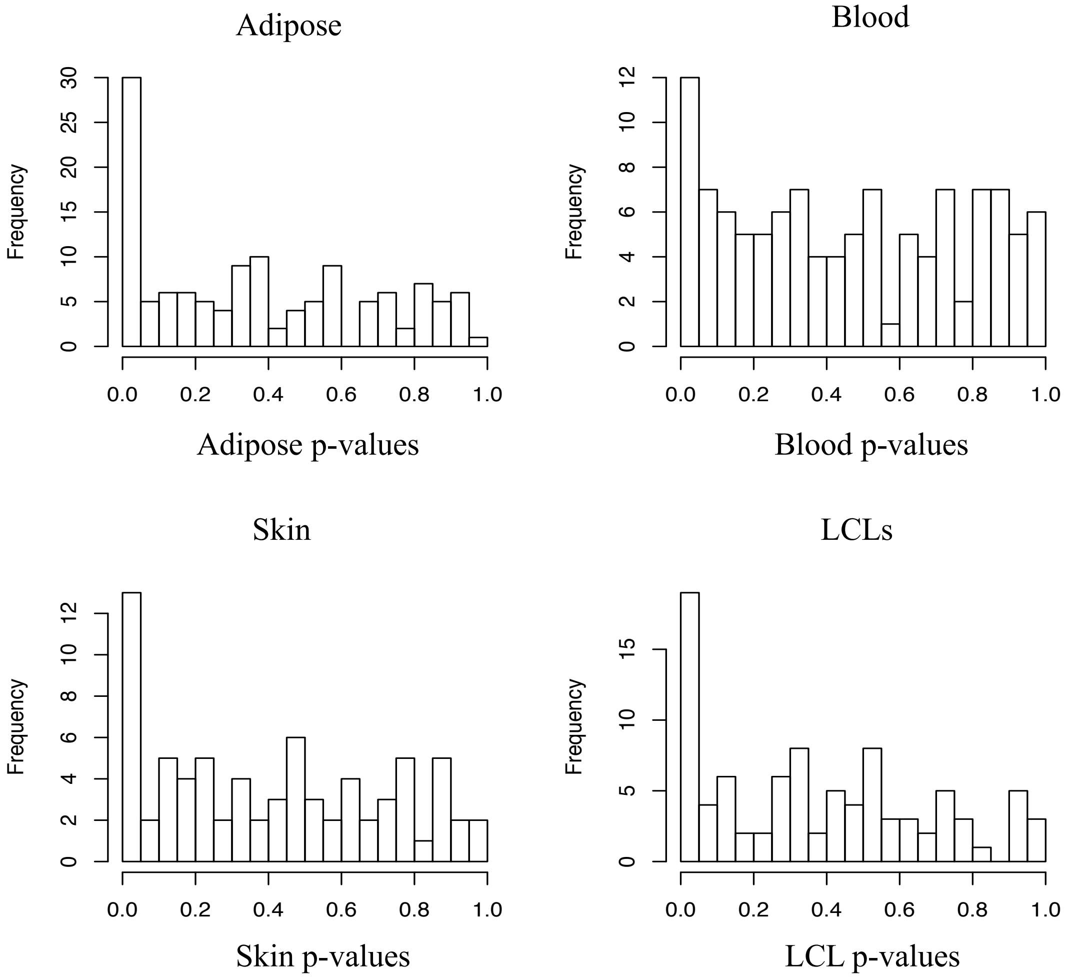

Figure S6: GxBMI enrichment in main-effect eQTL datasets. Histogram of p-values of matched GxBMI Exon-SNP in main effect eQTL data. Largest enrichment seen for adipose eQTLs.

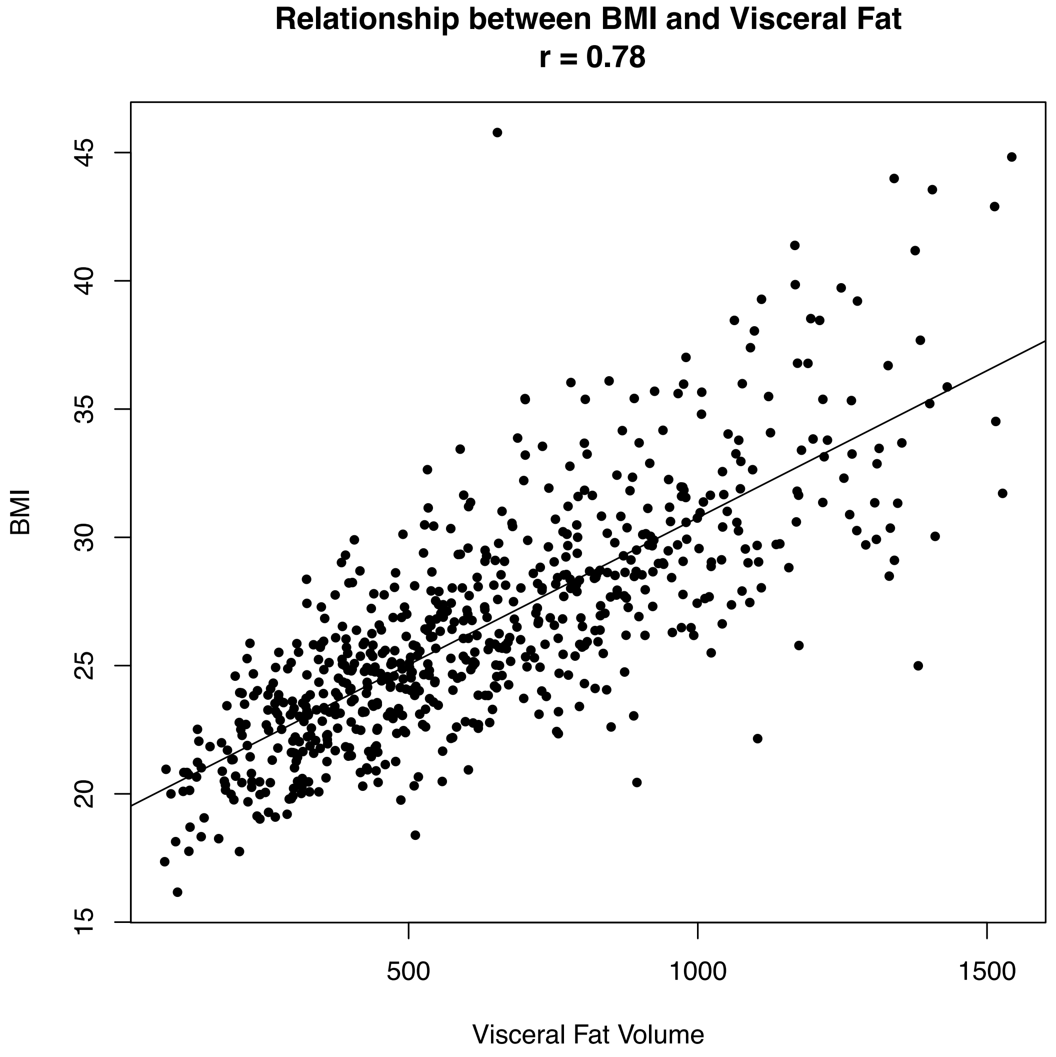

Figure S7: Relationship between BMI and Visceral fat volume, measured by Dual X-ray absorptiometry (DXA).

Gene	deCODE exon start(hg38)	deCODE exon end (hg38)	Beta	*P*-value	% coverage of TwinsUK exon
SPAG17	117,971,862	117,972,047	0.010	0.083	69%
CHURC1	64,932,137	64,935,366	0.011	8.5×10^{-4}	95%
CIDEA	12,262,824	12,262,969	-0.004	0.21	100%
PEPD	33,386,948	33,387,481	-0.014	4.2×10^{-6}	100%
*PHACTR3**	59,845,188	59,845,265	-0.014	0.005	4%
*PHACTR3**	59,774,242	59,774,490	-0.020	0.0001	100%
IFNAR1	33,349,388	33,349,543	0.002	0.58	100%
SCFD2	53,273,825	53,274,001	-0.006	0.26	100%
ADH1A	99,282,345	99,282,606	-0.0015	0.85	33%
ANXA5	121,667,996	121,668,527	0.001	0.52	93%
CAST	96,770,530	96,770,602	-0.004	0.053	4%
HLA-DQB2	32,758,849	32,759,131	-0.004	0.45	100%
ZNF117	64,989,946	64,991,036	0.0009	0.79	100%
ERV3-1	64,990,354	64,993,414	-0.0008	0.84	85%

Table S1: deCODE replication meta-exons. Percent overlap calculated based on length of exon in deCODE compared to that quantified in TwinsUK. *As two *PHACTR3* meta-exons were significant in TwinsUK and the lead TwinsUK meta-exon had an overlap of 4% with deCODE, the second 100% overlapping meta-exon was used for replication analysis.

Tissue	Samples	Samples with genotypes	# expressed exons	# expressed genes
**Adipose**	766	720	118,643	19,111
**Skin**	716	672	114,377	19.901
**Blood**	384	368	85,811	16,149
**LCLs**	814	765	116,529	18,229

Table S2: Number of samples, genes and exons quantified in each tissue.

Gene	SNP	Non-PEER Beta	Non-PEER P-value	PEER Beta	PEER p-value
HACL1	rs1464171	+	4.77×10^{-9}	+	6.20×10^{-8}
ALG9	rs3851570	-	2.35×10^{-8}	-	0.016
SMG6	rs113368712	-	4.84×10^{-7}	-	0.00042
GAA	rs35662778	+	6.64×10^{-7}	+	3.46×10^{-7}

Table S4: non-PEER and PEER GxBMI. All non-PEER GxBMI are significant ($p < 0.05$) in the PEER corrected analysis. *ALG9* which exhibits a multi-gene *trans*-acting effect is diminished significantly when correcting for latent factors, reinforcing the idea that PEER removes biological *trans*-signals.

Type	Pathway	Corrected P-value
Metabolic	LXL/RXR activation	6.05×10^{-3}
	Uptake of cholesterol	9.06×10^{-5}
Immune response	Antigen presentation pathway	2.0×10^{-4}
	Adhesion of blood platelets	2.55×10^{-5}
	Phagocytosis of fibroblasts	2.57×10^{-4}
	Quantity of macrophages	5.77×10^{-3}

Table S8: Genes regulated by adipose GxBMI effects are enriched for metabolic and immune response processes.
